# Living on low-incomes with multiple long-term health conditions: A new method to explore the complex interaction between finance and health

**DOI:** 10.1371/journal.pone.0305827

**Published:** 2024-06-26

**Authors:** Olga Biosca, Enrico Bellazzecca, Cam Donaldson, Ahalya Bala, Marta Mojarrieta, Gregory White, Neil McHugh, Rachel Baker, Jonathan Morduch

**Affiliations:** 1 Yunus Centre for Social Business and Health, Glasgow Caledonian University, Glasgow, United Kingdom; 2 Department of Management, Economics and Industrial Engineering, Politecnico di Milano, Milan, Italy; 3 National Centre for Epidemiology & Public Health, Australian National University, Canberra, Australia; 4 School of Law and Social Sciences, Oxford Brookes University, Oxford, United Kingdom; 5 National Centre for Social Research, London, United Kingdom; 6 Wagner Graduate School of Public Service, New York University, New York, NY, United States of America; University of Rome Tor Vergata: Universita degli Studi di Roma Tor Vergata, ITALY

## Abstract

People on low-incomes in the UK develop multiple long-term health conditions over 10 years earlier than affluent individuals. Financial diaries -new to public health- are used to explore the lived experiences of financially-vulnerable individuals, diagnosed with at least one long-term condition, living in two inner-city London Boroughs. Findings show that the health status of these individuals is a key barrier to work opportunities, undermining their income. Their precarious and uncertain financial situation, sometimes combined with housing issues, increased stress and anxiety which, in turn, contributed to further deteriorate participants’ health. Long-term health conditions limited the strategies to overcome moments of financial crisis and diarists frequently used credit to cope. Restrictions to access reliable services and timely support were connected to the progression of multiple long-term conditions. Models that integrate healthcare, public health, welfare and financial support are needed to slow down the progression from one to many long-term health conditions.

## Introduction

The increase of individuals living with two or more long-term health conditions has become one of the main challenges facing health and care systems around the world [1]. Living with multiple long-term health conditions has important implications for individuals, carers and societies, as well as for health systems and health care delivery [2]. These conditions have no cure but can be managed with drugs and/or other treatments and may be related to physical and/or mental health [2, 3]. The development of more than one long-term health condition is a complex process in which multiple underlying mechanisms interact at different levels. Age and lifestyle risk factors are well-known determinants of long-term health conditions, however other broader determinants, such as socio-economic deprivation, also play a fundamental role and are less understood [4–9]. Studies in the UK have shown that people living in the most deprived areas develop multiple long-term health conditions 10–15 years earlier than those in the most affluent [10]. Researchers and governments alike have, in recent years, increasingly expanded their focus beyond mortality to morbidity [9–13]. Extending healthy life expectancy and reducing inequalities has now become a policy objective in high-income countries such as the UK [13]. However, this is a difficult task and a deeper understanding of how socio-economic deprivation and health relate to each other is crucial. Developing an evidence base to inform the design of multifaceted income-enhancing and income-stabilising initiatives, such as responsible credit, could slow down the progression “from one to many” long-term health conditions in populations, beyond the elderly, in whom these conditions are more prevalent [14–16].

Having a long-term health condition has been linked to lower participation in the labour market and poorer economic status, with these effects being substantially worse for those with multiple conditions who are even less likely to be employed and, consequently, have drastically lower incomes [8, 9]. Income, and its unequal distribution, is recognised as a key social determinant of health (SDH) [17–19]. SDH are non-medical factors that influence health outcomes or, in other words, the conditions in which people are born, grow, work, live, and age [20]. Despite the established link between income and health [11, 17–19], the pathways and mechanisms through which income can affect health remain relatively unexplored and even more so when examining the progression of long-term health conditions [4, 21, 22]. This is mostly due to the multifaceted nature of personal finances and the interrelatedness of the different SDH. Existing research on income and the development of multiple long-term health conditions has been focused on total household wealth, and individual, household or area-level income measures [4, 21]. Recent voices have argued that a more nuanced view of personal finances is needed. For example, the US Healthy People 2030 Agenda considers ‘economic stability’ instead of income as a SDH. The concept of economic stability goes beyond income to include expenditure, debt, employment, support and medical bills [23]. Scholars have also advocated for the use of less traditional, more comprehensive, measures such as discretionary income, i.e. the amount of money left over at the end of the month, financial health, which includes the ability to meet basic needs, save and build wealth, or financial capability as a SDH [15, 24–26]. We build upon these arguments to comprehensively explore how the different financial aspects of individuals’ lives link to health.

Financial difficulty, including use of highly-priced credit, has been linked to detrimental impacts on stress, anxiety and physical health [28–31]. Although having access to credit, particularly in crisis situations, is also recognised as having potential to impact positively on health [32–33]. Debt type matters for health too [34]. However, little is known about the challenges faced by those in difficult financial situations and their coping strategies whilst simultaneously managing multiple long-term conditions.

This study explores this gap using financial diaries—new to public health–that enable unique, multidimensional, insights into the lived experiences of study participants. By closely following the lives of participants over a 6-month period, financial diaries allow for longitudinal, in-depth data to be collected. Data was obtained not only on income levels but changes in income and expenditure over time, savings and assets, how individuals cope with (often-unexpected) changes to their incomes and expenditures, and the range of financial products used (e.g. credit) to stabilise income and consumption. This study is focused on people already diagnosed with at least one long-term health condition living in deprived communities in the inner-city London Boroughs of Lambeth and Southwark. To permit a focus on finance and health, part of the sample was drawn from customers of Fair Finance, a local microcredit provider. Ultimately, our aim is to inform the design and implementation of income-based programmes focused on slowing the progression of ’one to many’ long-term conditions.

## Methods

### Setting and study population

One in five (i.e. 140,000) residents of Lambeth and Southwark lives with at least one long-term health condition, and over 19,000 live with three or more conditions. Lambeth and Southwark are two of the most densely populated boroughs in the UK and the population is largely young on average (50% aged 35 years or under) [5]. There is also a complex ethnic mix in both Lambeth (65% white) and Southwark (61% white), particularly when compared with the UK as a whole (88% white). Approximately one third of residents in each borough were born outside of the UK with the largest shares coming from the European Union and Central and South America. In 2019, close to half of all Lower layer Super Output Areas (LSOAs) in Lambeth and Southwark were among 30% of the most deprived in the UK [35]. Both Lambeth (28%) and Southwark (25%) have higher levels of poverty when compared to the four UK countries which on average range from 16% to 22% [35, 36]. In addition, within-borough income inequality is high and persistent. For example, there is a £15,000 difference in average household income between Tulse Hill and Herne Hill/Dulwich Park, two neighbourhoods less than a mile from each other in the Boroughs. Health inequalities between the more and less affluent areas are equally stark in Lambeth and Southwark. For example, there is a 17-year gap between Tulse Hill and Herne Hill/Dulwich Park in female healthy life expectancy, i.e. the average number of years a woman might expect to live in good health in their lifetime [37]. We purposefully sampled a small group of residents (n = 21), reflecting a diversity of lived experience in terms of age, ethnicity and household composition. Selected participants needed to have been clinically diagnosed with any long-term health condition that had lasted, or was expected to last, longer than three months. Arthritis, diabetes, pulmonary conditions, and depression were all common examples in our sample, capturing the most substantial conditions from a population-based epidemiological perspective [2, 5]. Recruitment was undertaken in English, Portuguese, Spanish and French as these are the most commonly spoken languages in these London Boroughs. To reach ‘seldom-heard communities’, referral sampling through local organisations was used, as well as posters and leaflets and an online recruitment questionnaire.

Due to difficulties in capturing accurate and reliable household income for this particular group, we took the conservative approach of considering low-income individuals to be those on means-tested benefits. An exception was made with one participant who was receiving a state pension. Her level of income was below the Minimum Income Standard in London in 2017. Additionally, we sought to ensure the group comprised users and non-users of unsecured debt including responsible microcredit, i.e. regulated, affordable small loans to financially excluded people who pay a poverty premium to access financial services. Twenty-one working-age individuals completed financial diaries over a period of four to six months each. Based on their long-term conditions and debt status, eight of them were sub-sampled for an in-depth interview to explore their money management experiences whilst simultaneously managing multiple long-term health conditions.

Recruitment and data collection took place between 27^th^ May 2019 and 28^th^ February 2020. Participants provided written informed consent ahead of their participation in the baseline interview and ongoing oral consent, voice recorded, at the start of every monthly interview. Ethical approval was granted from Glasgow School for Business and Society Ethical Committee [EC014], Glasgow Caledonian University.

### Financial diaries

Financial diaries are systematic records of all daily income and expenditure transactions, aimed at understanding money management strategies over time [38]. Originally developed to explore the financial behaviour of individuals and households living on low-incomes in countries such as India, Bangladesh and South-Africa, this method has recently been adapted for use in high-income contexts such as the United States [39], Canada [40] and the UK [41]. In this study, financial diaries have explored in-depth not only money management and financial decisions but also if and how these are linked with mental and physical health.

Financial diary data were collected in monthly diary-interviews that took place in participants’ homes, or in public spaces. Data were elicited at individual, not household, level to focus on the financial behaviour of those living with long-term health conditions. In total, we conducted 131interviews which lasted between one and two hours. These interviews were in principle conducted monthly but they were more frequent for participants with health conditions that prevented them from recalling and/or recording accurate data. The data collection period with each participant varied from four (n = 4) to six months (n = 17). A total of 118 monthly diary entries were collected.

In line with seminal financial diaries research [38–41], the term ‘diaries’ was used to reflect the high-frequency of data collection and not the diarists logging transactions themselves. Researchers systematically recorded almost 9,000 diarists’ income and expenditure transactions. As shown in Table 1, the predefined variables captured for each transaction were: purpose, amount, direction of transaction, method of payment, person/organisation, and ‘additional comments’ to capture rationales and other important characteristics of the transactions that were not included elsewhere. We classified every transaction into a standard category for analysis. This information on transactions was used for prompts to generate qualitative data in every monthly interview–‘event records’–in relation to participants’ lives, social networks, health and life events, and ‘moments of crisis’ or ‘cliff-edge moments’. These are particular situations in diarists’ lives where a sudden change leads to multiple, immediate and potentially very serious problems.

**Table 1:** Financial diary data collected.

Variables	Description
Date	Date of transaction
Purpose	Item of transaction
Amount	Recorded to the nearest £
Category	Accessories; Benefits; Bills; Business; Celebration; Charity; Children; Clothing; Communications; Education; Entertainment; Financial; Gambling; Gift; Groceries; Health; Household; Housing; Income; IT; Miscellaneous; Service; Taxes; Transport.
Direction of transaction	Outflow Inflow
Method of payment	Cash; Debit card; Credit card; Electronic financial transfer; Cheque; PayPal
Person/organization	Person/organization with whom the transaction took place. It was only collected for financial transactions related to: credit, savings, insurance and pensions.
Additional comments	To note rationales and inconsistencies in the data. For example, the reason for getting a loan.

The Short Form Survey (SF-12), a standardised health survey, was also elicited in monthly interviews. The SF-12 is a general health questionnaire used as a quality of life measure to assess the impact of health on an individual’s everyday life. The SF-12v1, used in this study, contains 12 questions on eight different health dimensions: general health, physical health, limited physical function, physical pain, vitality, mental health, limited emotional function and social functioning. The scoring of the responses gives two summary measures: the Physical Component Summary (PCS) and the Mental Component Summary (MCS) [42].

To maintain the quality of the diary data: (a) participants were sent weekly reminders to minimise recall bias; (b) diarists’ bank statements and receipts were cross-checked with reported transactions; (c) inconsistencies in income and savings against expenditure were tracked and addressed on subsequent visits to the diarist; and (d) data on cash-in-hand and savings were explored. While data were collected monthly, the research team maintained weekly/bi-monthly contact with diarists.

Participants also completed a baseline and final survey, administered respectively on the first and last diary-interviews, to collect information on socio-demographic characteristics, personal finances, and health and wellbeing. These questionnaires can be found in S1 File. This supporting information file (S1 File) also includes the topic guide for the semi-structured interviews, conducted with a purposively chosen subsample of eight diarists, to further explore their lived experiences managing particular long-term health conditions alongside debt financial products.

### Data analysis

A mixed-methods approach was used to analyse diary data [38–41]. The quantitative data from the financial diaries and the qualitative data from the event records and in-depth interviews was analysed concurrently and integrated for interpretation. Income and expenditure were analysed through monthly cash flow statements or budgets for each participant to identify trends and outlier transactions. This was compared to the analysis of the individual monthly trend of the subjective health measure (SF-12v1). Qualitative data was analysed through inductive reflexive thematic analysis, using NVivo 12 [43, 44]. The financial diary monthly interviews were recorded, when consent was given, but not transcribed in full. The researchers filled-in detailed event records after each diary-interview in which they could add field notes of their perceptions and assumptions during the interview. The lead author checked these event records for accuracy and meaning. The event records were analysed by the researchers and the emerging data-driven themes were used as a basis to develop the topic guide for the eight in-depth interviews. The in-depth interviews were recorded and transcribed. All data was coded with the supporting principles of constant comparison to ensure the robustness of the emerging themes [38, 43]. Themes were developed from and strongly linked to the data and understood through the researchers’ individual experiences, social and cultural contexts [44]. The diversity of the research team in terms of nationality, ethnicity and professional or academic backgrounds enriched the reflection on data interpretation and theme development. Pseudonyms have been used to maintain diarists’ anonymity.

Multiple long-term health conditions are more prevalent in underrepresented and minority population groups [5]. To facilitate reaching out to groups with limited English proficiency we devised recruitment materials in four languages (English, Portuguese, Spanish and French) and undertook research in Spanish (n = 4) and English (n = 17). Two members of the team were native Spanish speakers and proficient in English, French and Portuguese. They translated all recruitment materials and research instruments from English. The data collected in health event records, field notes and interview transcripts were translated to English by the researchers who collected it so that they could be analysed alongside the rest of the data by the full research team. For challenging translations, they sought advice from the full research team and/or the participants from which the data had been collected. The translations and coding of the translated data were reviewed by both researchers-translators to identify any altered or lost meaning [45].

## Results

Tables 2–4 show the final samples of diarists and interviewees. The 21 diarists, ranging from 27 to 67 years of age, were all managing at least one and up to eight long-term health conditions which also made them financially vulnerable [46]. All, except for one, were women and not working. Sixteen diarists were from ethnic minorities, seven were not UK born, four did not speak English, and all, except for one, were receiving means-tested benefits, indicating that their income and assets were low. The eight diarists sampled for in-depth interviews are indicated with an asterisk in Table 4. They all had lived experiences of managing a range of long-term conditions and financial shocks, they were all female and not at work, and they were all using at least one form of debt, preferably a loan from an affordable lender.

**Table 2:** Sample of diarists.

	Total
Referred and approached	58
Recruited for diaries	29
Drop-outs (before 4^th^ diary)	8
Total diarists*	21

* Includes 4 drop-outs after the 4th diary whose data could still be included in the study.

**Table 3:** Socio-demographic characteristics of the sample.

	Total	
	N	*%*
*Age groups*		
18–35	3	14%
36–45	5	24%
46–55	6	29%
>55	7	33%
Female	20	95%
Non-British background	16	76%
Full/part-time employment	1	5%
Welfare benefits (means-tested)	20	95%
Registered as disabled	11	52%
*Household composition*		
Lone parent w/ dependent children	8	38%
Couple w/ dependent children	2	10%
Couple with no children	1	5%
Single	2	10%
Separated/ Widowed	7	10%
Family abroad	1	5%
*Health status* *		
One long-term health condition	2	10%
Two long-term health conditions	5	24%
Three or more long-term health conditions	14	67%
Total diarists	21	100%

*As per baseline questionnaire

**Table 4:** Main characteristics of the financial diarists.

Name	Gender	Age	Origin^1^	Ethnic minority	Employment situation	Health conditions^2^	Registered disabled	Means-tested benefits	Disability and sickness benefits	No. of financial providers
Anya*	Female	46–55	UK	Yes	Permanently sick/disabled	COPD; Hypertension; Diabetes	Yes	Yes	Yes	3
Andrea	Female	36–45	LAC	Yes	Statutory sick pay	Arthritis; GI disorders; Asthma; Knee, shoulder, neck and spine injuries; Epilepsy; Thyroid problems; Anxiety and depression	No	Yes	No	2
Ayleen*	Female	>55	UK	No	Permanently sick/disabled	Arthritis; Musculoskeletal disorders; Anxiety and depression	Yes	Yes	Yes	6
Claire	Female	36–45	UK	No	Permanently sick/disabled	Anxiety and depression; PTSD	No	Yes	Yes	0
Cyra*	Female	18–35	UK	Yes	Permanently sick/disabled	Keratoconus; Anxiety and depression; Schizophrenia and other psychoses; Bipolar disorder	Yes	Yes	Yes	6
Leona	Female	46–55	UK	Yes	Unemployed and available for work	Arthritis; Prediabetes; Blepharitis	No	Yes	No	1
Carolina	Female	46–55	LAC	Yes	Permanently sick/disabled	Arthrosis; Fibromyalgia; Anxiety and depression	No	Yes	Yes	1
Luisa	Female	18–35	LAC	Yes	Unemployed and available for work	Anxiety and depression	No	Yes	No	3
Cassandra*	Female	46–55	LAC	Yes	Looking after family/home	Lupus; Hypertension; Prediabetes; Anxiety and depression	No	Yes	Yes	3
Hilda	Female	>55	African	Yes	Wholly retired from work	Arthritis; Hypertension; Cataracts; Anxiety and depression	No	No	No	1
Anna	Female	>55	UK	Yes	Wholly retired from work	Diabetes; Hypertension; Chronic back pain; Muscoloskeletal disorders; Anxiety and depression	Yes	Yes	Yes	1
Juliet*	Female	>55	UK	No	Permanently sick/disabled	Arthritis; COPD; Brain cysts	Yes	Yes	No	2
Madeleine	Female	>55	UK	Yes	Unemployed and available for work	Hypercholesterolemia; Hypertension; Knee, shoulder, neck and spine injuries; Anxiety and depression; PTSD	No	Yes	Yes	0
Mayowa	Female	>55	UK	Yes	Employee in part time job	Hypertension; Drepanocitosis	Yes	Yes	Yes	2
Sofia*	Female	36–45	LAC	Yes	Permanently sick/disabled	Hypertension; Diabetes; Chronic back pain; Anxiety and depression; Schizophrenia and other psychoses	Yes	Yes	Yes	6
Sarah	Female	36–45	UK	No	Unemployed and available for work	Arthritis; Asthma; COPD; Anxiety and depression	Yes	Yes	No	1
Teresa	Female	36–45	LAC	Yes	Unemployed and available for work	HIV; Chronic back pain; Anxiety and depression; PTSD	No	Yes	No	1
Daliya*	Female	18–35	UK	Yes	Looking after family/home	Nystagmus; Anxiety and depression; Bipolar disorders; Prediabetes	Yes	Yes	Yes	6
Carla	Female	>55	UK	Yes	Permanently sick/disabled	Arthritis; Fibromyalgia; GI disorders; Hypertension; Prediabetes; Knee, shoulder, neck and spine injuries; Glaucoma; Osteoporosis; Anxiety and depression	Yes	Yes	Yes	4
Shannon*	Female	46–55	UK	Yes	Permanently sick/disabled	Knee, shoulder, neck and spine injuries; Schizophrenia and other psychoses	Yes	Yes	No	2
Charles	Male	46–55	UK	No	Permanently sick/disabled	Anxiety and depression	No	Yes	No	1

* Diarists also sampled for in-depth interviews

^1^ UK: United Kingdom; LAC: Latin America and the Caribbean.

^2^ COPD: Chronic obstructive pulmonary disease; GI: Gastrointestinal; PTSD: Post-traumatic stress disorder; HIV: Human immunodeficiency virus

Recruitment and retention were challenging due to the level of intensity and sensitivity of the data collected. Women were more likely to be referred and to meet the selection criteria, given higher prevalence of long-term health conditions and higher proportion living on low-income [47] and were also more likely to engage in the recruitment process. In addition to this, retention of working age males was challenging. This was mainly due to the time restrictions imposed by their working schedules, which usually involved working long irregular shifts and/or working multiple jobs.

For most diarists, complex financial lives and health status were inextricably linked. While 18 out of 21 diarists perceived that their health impacted their finances, all perceived to some extent that their finances had an impact on their health.

Analysis of the diaries plus the interviews revealed several recurrent themes and subthemes that are presented in Table 5. Each of these are evidenced in more detail in the forthcoming sub-sections.

**Table 5:** Analytical themes and sub-themes.

Themes	Subthemes
Employment and long-term health conditions	Need for flexibility Precarious working conditions Job insecurity Leaving the labour market
Income and health	Welfare Limited options to increase income Coping with life events Income, mental and physical health connection
Prevalence of finance and formal credit	Use of financial services Financial decision-making Debt Access to financial products and health
Responsible microcredit and the stress of debt	Microcredit as an alternative form of debt Microloan design and health
Reliance on debt from relatives and doorstep lenders	Informal borrowing experiences Experiences with doorstep lenders
Housing	Housing conditions Access and moving
Barriers to accessing public services	Health as a barrier Language Access routes Siloed services Processes difficult to navigate Lack of awareness and support

### Employment and long-term health conditions

Employment (or lack thereof) emerged as a strong determinant of the relationship between finances and health. At baseline only one diarist was working, nine were unemployed, 10 were permanently sick or disabled, and one was retired. Long-term health conditions were an important barrier for diarists not being able to access formal employment, even if they wanted to. Anya eventually stopped working due to her COPD and worsening diabetes:

“*People might look at you and think*, *“God*, *well you could go out and get a job*,*” and I always say*, *for me … you might look at me today and think*, *“Yes*, *she’s capable of working*.*” Tomorrow morning*, *I could get up and I’ve got the oxygen on all day*, *you know*, *and for me*, *an employer doesn’t want someone who can come in three days a week*. *They want someone for five days*.*”* (Anya).

In addition to absenteeism related to their health conditions, some diarists were having up to eight medical appointments per month, which would require exceptional flexibility from employers to accommodate.

The combination of low-paid jobs and lack of support with childcare and other caring responsibilities was a significant barrier to working:

“*Why did I bother going out to work*? *I was thinking I’m worse off than I was before*. *I was having problems having someone to pick the kids up from school*. *And I’d get a phone call if they hadn’t been picked up*, *because I was doing 10 hour shifts*.*”* (Shannon)

Informal work (e.g. as cleaners or waitresses) occurred amongst some diarists during the period of the study (see S2 File 3, Luisa, and 4, Andrea). However, working conditions led to these diarists having to stop, with one at the point where she might be unable to work again. Working conditions and job insecurity frequently prevent people from taking time off and being able to recover after a health shock:

“*One day I was at work and it was like the room was spinning and I fainted (…) So they took me to Emergency and they said my blood pressure was low*. *Then I couldn’t take much time off because if I didn’t work I didn’t get paid*. *So I was back at work the next day*, *I remember*.*”* (Shannon)

Some diarists were volunteering as they felt it helped to keep them occupied and was a way to socialise. For those who could no longer work, adapting to the new situation had not been easy:

“*I haven’t worked for ten years now*, *and it took me a long time to get my head around that*. *I’ve had counselling*, *having to deal with it*, *because I have to keep myself occupied now*.*”* (Anya)

Finally, others like Ayleen feel that if they had been given better medical treatment, they would have never needed to stop working in the first place:

“*I think because the NHS is so poorly funded*, *I think if people like myself would have had an operation*, *say and their back fixed*, *I would have been able to work an extra 20 years and been able to contribute to taxes and everything else*. *And to help myself and not to be a burden to the NHS*, *then afterwards by having to go to hospitals*, *physios*, *by having a mental illness*, *all that sort of thing*. *I think it’s really the physical illness has got an awful lot to do with finances in a sense*, *in that way*.*”* (Ayleen)

### Income and health

Long-term health conditions are an important barrier to accessing jobs and therefore to securing a reliable, stable, and sufficient income. All research participants were in receipt of at least one type of welfare benefit payment. Most diarists received a combination of benefits (n = 20), including means-tested and sickness and disability benefits. However, in some of these households, there were times during this study when these payments were not sufficient to cover all essential expenditure, not only due to the costs associated with living in London, but also because some diarists experienced unexpected cuts to, and interruption of, the benefits themselves (see S2 File, for example case 1, Anya). Healthy households generally have access to a wider range of options (e.g. working more hours) when money is needed for an emergency. However, for those in the sample such options were more restricted. Most diarists found that their financial life was easier when they were working, before their conditions worsened. Diarists found saving extremely hard which, in turn, affected their resilience to financial events such as, for example, a broken fridge (again, see S2 File, Anya’s case). The balance between income and expenditure was delicate. We observed how diarists had to carefully prioritise between different types of essential expenditures such as appliances and repairs, bills, council tax, housing, legal expenses, childcare, and food.

Constant financial management, coping with emergency expenditure and prioritising expenses while leaving others unpaid was stressful and, at baseline, not only a source of worry for most (76% of the sample), but also a ‘way of life’:

*“You haven’t got a clue what it’s like to sometimes go hungry*, *if you know what I mean*, *or feel as if you’ve got nothing and you haven’t even got a penny in your purse and you can’t go out and get a pint of milk because you’ve got to have black tea for the day*, *sort of thing*. *So you sometimes feel a bit like that*, *that it does affect your mood*, *it does affect your way of life*, *in a sense*.*”* (Ayleen).

For lone parents or those with limited support from their partners, managing such financial hardship was even more difficult and stressful, and represented a threat to mental health. For example, during one of our qualitative interviews, Cyra told us:

*“It’s hard and it’s a struggle to raise two boys financially it’s a drain*, *a constant drain*, *I feel constantly drained*. *(…) They’re constantly needing something*, *they ruin their clothes*, *it’s just a nightmare and that is what I find very expensive and food as well*.*”* (Cyra)

Three individuals out of the 21 in the sample reported frequent gambling (for example, online casino, online bingo and scratch cards). Two of them reported gambling as a financial strategy to try to cope with expenses that they could not afford, particularly when they could not access credit. Diaries showed moderate sporadic gambling for many others. Gamblers in the sample, due to mental health problems and medication, found it difficult to keep track of how much they were spending and the consequences that losing the money would have on their capacity of buying essentials.

Diarists perceived a direct connection existed between income and, in particular, mental health; low, and at times, uncertain income was perceived as contributing to worsening mental health, and subsequently their other long-term health conditions. Some diarists were able to articulate the complex relationship in which finances were connected to mental health and, in turn, physical health:

“*I think sometimes if you’re financially okay*, *your health seems to be a wee bit better*, *you seem to be happier*. *So mentally*, *you’re a little bit more happy and when you’re financially strapped and you haven’t got enough money*, *it makes your mood a lot less … it makes you more depressed*. *So you feel more depressed and so therefore your ailments seem to be more prominent*, *I think in some senses*.*”* (Ayleen)

### Prevalence of finance and formal credit

Due to the combination of low income, financial uncertainty and insecurity, finance plays an essential part of diarists’ lives. We observed that diarists made extensive use of financial services to help them smooth their consumption patterns at difficult times when their income did not match their expenditure. Finance-related transactions—those including credit, savings, insurance, and other financial services—were the second most frequent overall (11 percent), after groceries (17 percent) and closely followed by entertainment (10 percent). Most of these financial transactions were related to credit and credit arrears, followed by insurance products (mainly related to housing).

On average, every month each participant was making around 12 decisions related to financial services such as taking out or paying back a loan or paying for insurance. Financial decisions are particularly complex and stressful; they deal with relatively large amounts of money, can have serious future implications, and are not easily reversed. For diarists, these decisions are even harder to make; urgency, poor mental health, and lack of control all combine to increase stress levels and anxiety.

Despite their financial vulnerability and their low incomes, diarists had extremely complex financial lives. Everyone in the sample, except two diarists, was managing at least one loan (90 percent, n = 19). More than half of the sample (57 percent, n = 12) were simultaneously using at least two types of loans during the study. Over the data collection period, study participants were using two credit products on average and four diarists were each found to be simultaneously managing six loans.

Only one third of diarists had reported, on the first meeting, having £1000 or more saved to cope with a financial emergency. Their low incomes and unpredictable variations in income and expenditure meant that it was especially difficult to save, making borrowing the only option to stabilise consumption over time.

All the loans that were taken out just before and during the research period were to cover essential needs. Almost all were for housing-related items; some were for refurbishment and decoration and others to urgently replace broken white goods. One loan was used to pay for a trip abroad to visit family after seven years of not seeing them and to introduce children to their grandparents in the Americas. An important category of emergency expenditure for non-UK citizens was obtaining visas and other Home Office related expenses. These expenses could be particularly destabilising; they were not planned, they were difficult to control or postpone, they came with associated expenses such as paying for legal advice, they were of relatively large amounts and the consequences of not paying were serious and immediate.

Due to recently having been able to work, some diarists could access mainstream-priced financial institutions (banks and credit unions) and products (overdrafts, credit cards, mortgages) first accessed when more financially secure. However, the main financial product they could access during the study was high-cost credit, such as catalogues, store cards and doorstep loans. The wide range of debt instruments used by the 19 diarists in the sample who had at least one current loan are shown in Table 6.

**Table 6:** Debt instruments.

Debt instruments	Total *(n = 19)*
Relatives and friends	12
Microcredit	7
Credit card	6
Budgeting loans	5
Doorstep	5
Catalogue	4
Overdraft	3
Pawn	3
Credit union loans	2
Store card	2
Rotating Savings & Credit Associations	1
Bank loan	1
Mortgage	1
Total	52

Financial inclusion in this sample can be a double-edged sword because individuals, who were once financially secure and previously working, experience changed circumstances after being diagnosed with a long-term condition. For example, Hilda, a 67-year-old woman, is the only diarist who has a mortgage in the sample. Having been made redundant, as her health conditions had worsened, and no longer able to afford her mortgage, her children now make the payments as she did not want to give up her house. Bank overdrafts are another example; some diarists have kept the overdraft limits they could access when they were healthy and have found difficulties reducing the size of this debt (see S2 File, case 2 Sofia). Most diarists using mainstream financial products had already taken out loans when their circumstances changed. The repayment of these loans, with instalment amounts corresponding to what participants could afford in a different situation, often have to be made from diarists’ benefit payments. This was not always possible and has been identified a source of further stress.

### Responsible microcredit and the stress of debt

A ‘safer’ strategy for others in the sample was to borrow from responsible finance providers such as community development financial institutions (CDFIs) and credit unions. These organisations provide access to affordable credit and financial capability in a fair and transparent way. In the past, Anya borrowed for her grandfather’s funeral, choosing a doorstep lender. During the loan period, interest rates kept rising. It took her two years to clear the debt: *“After*, *I said I’d never go back*.*”*.

Responsible microcredit was one such alternative that diarists could access to cover for emergencies when the amounts required exceeded the lending capacity of their informal networks or they chose not to ask them for help. Out of the seven borrowers that were using it, most were satisfied as it met their needs (small, fast, flexible, short-term, and affordable). The loans offered from Fair Finance had, in most cases, enabled them to swiftly replace broken white goods such as a new fridge or a washing machine. Borrowers indicated that they valued staff members that walked them through the process and how this process was straightforward and adapted to their health problems. They mostly appreciated the accessibility of the loans:

“*I think it would help so much because they don’t look at your credit history, they look at it but they don’t say because you don’t have a good history or you don’t have a history at all, they’re not going to lend to you, they at least give everybody a fair chance, do you know what I mean?” (Daliya)*

Borrowers also valued accessibility, affordability and that repayment was adjusted to when the benefits were paid so that the money was always in the bank when it was due, which reduced stress. Whilst responsible loans provided an alternative to high-cost subprime lenders, they (as with any type of debt) were reported to work better for those diarists who were more in control of their finances and, more generally, their lives. However, for borrowers with more advanced and more severe conditions, particularly mental health related, the microloan could become one more debt in a list of high-cost loans. Problems with loan repayment could add to the stress and anxiety experienced with other lenders contributing to a worsening of their already severe mental health problems.

Diarists who were worse-off in terms of their health benefited more from other forms of support such as budgeting loans. These interest-free loans, provided by the UK Government, were available to individuals who were accessing means-tested benefits. The loans were seen as a safe alternative to cope with emergencies for those diarists who were managing multiple and severe health conditions that impeded them being in control of their finances.

### Reliance on debt from relatives and doorstep lenders

Most diarists, however, were dealing with their financial insecurity by relying on informal loans with family, friends and employers. Migrant diarists in particular, but also others in the sample such as Charles, could not borrow from mainstream financial institutions because of unemployment, unstable employment, having a poor credit history or not having a credit history. Whilst some were more likely to be managing a portfolio of regulated subprime loans (pawn brokers, catalogue, doorstep, etc.) as well as loans from family and friends, others were managing their finances by relying solely on the latter.

Social networks were a main source of small, short-term, flexible, and interest-free loans. These characteristics make them valuable for diarists as they fit exactly with their needs. When we first met them, twelve diarists were indebted with family and friends. Diarists are not keen on asking their social network for money because they know that this puts pressure on loved ones that have their own financial struggles. Ayleen has an outstanding loan with a doorstep lender of £600 that she cannot pay back. She knows that due to that it is unlikely that other financial providers will lend her money. When she is strapped for cash, she asks her brother for help. Ayleen would prefer to get the money from a different source and she tells us:

“*It is very demeaning*, *I think in some senses*, *it makes you feel as if you’re some sort of leech*, *leeching off people for money*, *if you know what I mean*. *It makes you feel uncomfortable; it does make you feel uncomfortable*.*”* (Ayleen)

Charles, Ayleen, and most of diarists would prefer not to have to borrow from relatives and friends. They are scared that if they struggle to repay, their lenders will suffer as a consequence because their financial situation is also delicate. In Charles’ case, his brother borrows through his credit card to lend him the money. If Charles’ were not able to pay back, his brother might see his own credit score affected. However, in an emergency they have no other option but to rely on their loved ones for help.

Doorstep lenders were the most popular high-cost credit source in our sample and the majority of diarists had used them in the past. These fast, small and short-term loans were generally valued by diarists. However, diarists only borrowed from them at last resort, aware of their high costs and additional fees in case of default, as well as the threat of aggressive collection tactics.

### Housing

The precarious financial situation of the diarists meant that no diarists were able to afford a mortgage (Hilda, mentioned above, had a mortgage, but her children paid the instalments) and most diarists (n = 16) were unable to rent accommodation in the mainstream London market. All relied on housing benefits, usually put towards social housing provided by either local councils or housing associations. But in several cases we found that the low quality of the housing and the complex processes around accessing social housing or maintaining homes in good state had an impact on the diarists’ health:

*I had to go to the doctor … to take pills*, *because I was having a bad time*. *I was being evicted*. *I saw myself alone in the house*, *the dad of my children is*… *he went to his home country*, *and I prospect that is only one day left for them to kick me out [of the house]*. *I know they weren’t going to leave me in the street*, *but*… *whatever*… *whatever*… *they waited until the last day*, *there*, *in the council*, *there*, *in Lambeth*… *to give me one [house]*. (Cassandra)

Searching for a council house also emerged as a particularly challenging activity for some diarists already experiencing a hectic life full of health issues, health check appointments, re-assessment of working capacity and benefits and debt repayments. This also impacted on finances and health because, whilst trying to apply for social housing with no effective support from the council, diarists such as Andrea had to pay for poor-quality private housing that was over their budget and which was perceived as worsening their physical and mental health (see, for example, S2 File, case 4, Andrea). Some diarists were caught in a cycle of living in accommodation declared unsafe and posing further threats to health but still unable to move because of their health:

“*Where can I go*, *me*, *that I won’t get a contract because I am sick*, *who’s gonna give me a lease contract*?*”* (Carolina)

Despite some diarists having better experiences with housing associations, conditions of some homes still presented health threats. This comprised a lack of basic furniture, such as a bed, and of rails on the stairs, leaks, damp, and bad insulation.

### Barriers accessing public services

During the six months of interviews, many diarists experienced ‘cliff-edge moments’ in which sudden changes in their circumstances required different kinds of services and urgent support. All diarists in our sample, in particular those with mental health issues, struggled to access public services such as welfare benefits, healthcare, housing and social services. Diarists with poor mental health generally struggled to leave the house and were not comfortable repeatedly telling their story to strangers and those with physical long-term conditions experienced restricted mobility issues that limited their ability to access some services. Most diarists (n = 12), in particular those from ethnic minorities and those with limited English such as Andrea, faced challenges accessing and receiving appropriate support during the study. Some diarists reported that the organisations they were asking support from were not listening and so it did not make sense to make the effort to reach them. If they thought there were too many barriers, sometimes the diarists stopped seeking help. This applied to all the services that were discussed with them, from getting appointments for medical visits to accessing free legal advice. The consequences of not seeking help were serious and if this involved not accessing, in particular, welfare benefits or housing their situation could deteriorate fast.

The main example is accessing the NHS to get treatment for their conditions, particularly mental health in primary care settings. For example, Charles tells us:

“*It’s so difficult just to get an appointment with the doctor*. *You go there and they say there are no appointments on the system*, *but if you’re not seen*, *there’s not much you can do”*. (Charles)

Daliya, for example, told us about the complications in combining the different appointments and treatments for each of her comorbidities. She described how she had to discuss each of her conditions one by one with the doctors and at some point she realised that she had stopped getting treatment for one of them:

“*The GP kept calling me about other things*, *my other health conditions*, *yes*. *It’s like he kind of forgot about that one and I*, *myself*, *I forgot too*, *yes*.*”* (Daliya)

Daliya had to go to court for UK’s Department of Work and Pensions (DWP) to change their decision over her Personal Independence Payment (PIP), a benefit designed to support living with an illness, disability or mental health condition. She got the backdated payment but the process deeply affected her mental health. When asked about what could have made the situation better, she said:

“*I would say counselling*, *kind of*, *therapy*”. (Daliya)

Diarists referred to the need for more social support. Ayleen, for example, when asked what could have helped to slow down her conditions said:

“*Social help*, *in a sense*. *If you’ve got somebody coming in and seeing that you’re in a situation where say you haven’t got any milk or you haven’t got any sugar or you’re not too good with your finances at the time*, *then go and get me some help*, *sort of thing*.” (Ayleen)

Despite welfare generally being considered a relatively reliable and certain source of income, this was not necessarily the case for diarists. Diarists in the sample struggled to access the benefits system and there was a general lack of understanding about how some particular benefits worked and what to expect from changes of circumstances. Some diarists such as Luisa (see S2 File, case 3, Luisa), considered their employment decisions based on flawed assumptions of what the implications would be for their benefits. Reassessments and interruptions in the provision of some benefits and repeated variations in the amount of others (e.g. Universal Credit) directly affected the daily balance between income and expenditure in these households. Budgeting was also complicated by the scheduling of benefit payments. For example, some benefits had payments scheduled every four weeks rather than on the same date every month. This payment schedule did not align with dates of statutory expenses or more general bill payments which meant that in some weeks expenditure would substantially exceed income. Diarists would tell us:

“*No*, *if I’m skint*, *like this week is my week that I don’t get no money at all*. *(…) It’s tough luck*, *if I haven’t got money to go and buy something*, *it’s tough luck*.*”* (Juliet)

Luisa, Daliya and other diarists had struggled to access independent financial advice in the past even if they knew that they would benefit from it. In Daliya’s words:

“*I need somebody else’s intervention in helping me to budget but then I can do it*, *it’s just that I need that motivation to do it*, *yes*. *Sometimes if there’s anything I wanted to do with money but because of my learning disability I don’t understand some of the jargon that they throw at me*, *do you know what I mean*?*”* (Daliya)

When financial advice services had been used, some diarists did not find them useful. The main reason for this was that the diarists found it difficult to open up and share their real financial lives. This was due to a combination of shame and diarists distrusting the advisors. Cyra told us:

“*I haven’t been that honest… I haven’t really run into debt and spoken about it*, *I’ve spoken about other things*.*”* (Cyra)

The experiences of diarists with the Job Centre were also unsatisfactory. They felt the whole process was demeaning for them and that their particular health problems and the implications of their conditions were not considered. The same applied to housing providers where diarists felt that the staff they spoke to did not seem to understand their immediate needs due to their health conditions.

## Discussion and conclusion

Despite a substantial body of evidence around social and economic determinants of health and similarly around individual-level behavioural causes of health inequalities, these two areas of work rarely speak to each other [48]. Consequently, there is a lack of progress toward improving population health and reducing health inequalities. There is a recognised need for new approaches that link these areas together [16, 17]. As demonstrated by this study, financial diary methodology enables unique insight into how individuals respond to factors affecting them at a personal, community and more structural level. Financial diaries also shed light on the various and interacting pathways between socio-economic deprivation and multiple long-term health conditions, which remain mostly unexplained [14]. Beyond changes to income levels, financial diaries can identify innovative measures related to stability of income/expenditure, nature and frequency of shocks, and options and choices available to react to those shocks that give a more nuanced view of economic difficulty [15]. Financial products, such as savings or credit, are the main strategy to cope with everyday money problems and the characteristics of these products and how they are used can affect, in turn, the health and wellbeing of users. Financial diaries have also helped disentangle the perceived pathways between other social determinants of health that are closely related to socio-economic deprivation, mainly employment and housing, and identify where and why individuals experience challenges in accessing public service support. Adopting financial diaries more widely in the public health realm, in combination with other methods, could help enhance our understanding of how, for example, different forms of income-enhancing and income-stabilising initiatives could address health inequalities.

This work highlights how policies can address the complex interaction between employment, financial stability, housing, and access to essential services for individuals with long-term health conditions. Diaries revealed that employment was a key SDH for people with one or more multiple health conditions. Employment can have both positive and negative impacts on health, however it depends on the working conditions and flexibility towards accommodating individuals with different needs such as, for example, the need to attend numerous medical appointments [8, 9, 49]. Policies that promote good working conditions and healthy environments are particularly important for people just diagnosed with a long-term condition as these can prevent their health from deteriorating rapidly and support their income. Both broader policies, such as equal access to employment or minimum wage, as well as specific employment interventions to support vulnerable groups suggest that these could be a powerful tool to improve the health of particular population groups and reduce health inequalities [16].

For research participants, complex financial lives and health status were inextricably linked. Managing their livelihoods and multiple sources of unsecure and low income was exhausting and stressful. Psychosocial theories have linked sustained states of stress and lack of control to worse health and multiple health conditions, both through biological pathways and through encouraging health-compromising behaviours. Theoretically, any initiative or policy that reduces stressors (financial stress, parental stress, etc.) would help to slow down the progression of long-term conditions [14]. Employment and social security policies that ensure income levels are sufficient for decent standards of living are crucial for reducing health inequalities and, therefore, for individuals managing long-term health conditions. The benefits on health of policies related to income, wages and paid time off are consistently higher at lower income levels [50]. For example, recent evaluations of policies such as Universal Basic Income (UBI), where everyone receives a regular, unconditional cash payment, have been linked to a reduction of health inequalities, especially for the most precarious groups for which multiple long-term health conditions are more prevalent [51–53]. This is not only due to the additional income but also because of the stability and reliability, not linked to conditionality, of these type of payments.

Giving people adequate and reliable options to manage the delicate balance between income and expenditure is another way to reduce financial stress. This study confirms that dealing with insecure and/or low incomes by making extensive use of financial services to help them smooth difficult times requires an enormous amount of mental ‘bandwidth’ and aggravates stress [14, 54]. Financial decisions are hard because of the urgency, serious implications and large sums of money involved–on top of managing personal mental or physical health. However, these decisions are often not enough to overcome structural barriers or avert ‘cliff-edge’ moments when unexpected delays in income or essential expenditure cannot be met. Our study shows that mental and sometimes even physical health suffer as a result. Turning to family and friends for financial help does not work for everyone as it can depend on where you live and who you know: thus, financial, health and social inequalities can compound each other. Greater partnerships with financial advice and responsible lending organisations need to be encouraged and facilitated not only to help safeguard people with long-term conditions, particularly those experiencing mental health issues, but also for such organisations to recognise their role in sustaining and promoting better health and wellbeing. The Financial Conduct Authority could further facilitate this by prioritising support and ensuring that financial firms likely to have high numbers of customers on low incomes and multiple characteristics of vulnerability comply with regulations and standards, especially concerning the fair treatment of customers. Finally, innovative national policies such as Mental Health Crisis Breathing Space–legislation introduced in 2021 that gives debt protection to residents in England and Wales receiving mental health crisis treatment–are also important. Homes that are good for health are accessible, affordable, in good condition and secure, but most diarists lived in problematic housing situations. When maintaining health and work are a struggle and income from welfare payments fluctuates, securing a home is hard. People on low incomes often struggle to afford to rent their accommodation in London’s private rental sector. If they do, they have very little money left for the month. Housing benefits help, but are not enough. The low quality of private housing and the complex processes around accessing social housing can have a profound impact on people’s health [55]. Not only does poor quality housing exacerbate health issues, but the process of searching for social housing can be onerous and slow. The more challenging the process, the more of a mental health burden and strain this puts on people with long-term health conditions. Local authorities could engage more with the private rental sector through licensing schemes, strengthening tenant/landlord mediation, increasing enforcement and alignment of Discretionary Housing Payments with their population health strategies, securing more appropriate support for those with disabilities and long-term health conditions. Greater engagement with trusted local partners would also secure better accommodation for non-English speakers.

Access to health and social care, and other essential services, such as childcare and legal advice, are clear mediating mechanisms with the power to make situations better or worse. Many diarists experienced ‘cliff-edge moments’ in which sudden changes in their circumstances required different kinds of services and urgent support. Yet, each of the participants in this study struggled to access public services such as welfare benefits, healthcare, housing and social services. Accessing public services is often difficult for people with long-term health conditions–those with poor mental health can struggle to leave the house and are not comfortable repeatedly telling their story to strangers. People not fluent in English face additional challenges accessing and receiving appropriate support. To reduce these barriers, it is crucial to avoid introducing punitive measures which make life harder for individuals living in low-income communities, such as cutting or reducing welfare benefits.

Diaries show how costly accessing services is, in particular health services, even if the health system is free at point of use. This finding resonates well with the medical literature on long-term conditions that advocates for the need of person-centred care, rather than focused on single diseases, for a more coordinated and less disruptive holistic treatment [4]. In addition, improving the availability and accessibility of good quality and free primary health, social and child care will not only aid communities with low incomes, but also further cooperation and relationships between creditors, welfare providers, regulators and healthcare providers. In moving towards more comprehensive integrated care models, this could be achieved, for example, through social workers working in partnership with particular advisors or, simply by ensuring these services are provided more sustainably over time. Projects like the Deep End advice project in Glasgow or the Financial Shield project in London where financial, social security, housing and debt advice are provided alongside medical advice are possible examples [16]. Such integrated care models should encourage greater community voice in advising on service provision as well as further roll-out of social prescribing initiatives to support the most vulnerable in terms of health and financial vulnerability.

This study has some limitations. The financial diary sample only has one male participants and one employed individual out of 21 and the in-depth interview subsample (n = 8) is all female and not at work. Therefore, we must acknowledge that these findings mostly speak about the connection between finances and the progression of long-term health conditions for women who are no longer at work. The circumstances, pathways and mechanisms identified might be specific to this particular group. In addition, income and expenditure were self-reported and, though multiple processes were in place to preserve data accuracy and reliability, transactions might be missing from our records. Finally, microcredit borrowers in the sample were only using one product from a particular provider so we cannot assess if there were design and delivery features of affordable credit that were more directly connected with health. Future research should focus on if and how income-enhancing and, in particular, responsible microcredit initiatives, can improve health and reduce health inequalities at a population health level. Finally, it is worth noting that we completed data collection on February 2020, the month in which the first UK lockdowns in responses to COVID-19 commenced. This likely means that the lives we have described will have been made worse by the social and economic impacts of the lockdowns. In the meantime, what this study revealed, pre-pandemic, is the need to break down silos between organisations addressing inter-connected financial health, physical health, and mental health problems. If we are to move to a truly integrated health system, it is crucial that creditors, funders of debt services, regulators, and decision makers in welfare and healthcare systems recognise their role in furthering financial, mental and physical health of people with multiple long-term conditions.

## Supporting information

S1 File Instruments. Research instruments questionnaires and topic guide.
(DOCX)

S2 File Illustrative cases. The illustrative cases of Anya, Sofia, Luisa and Andrea.
(DOCX)
