# Supplementary material for: Living on low-incomes with multiple long-term health conditions: A new method to explore the complex interaction between finance and health
Source: PLoS One. 2024 Jun 26;19(6):e0305827. doi: 10.1371/journal.pone.0305827 (PMC11207141; doi:10.1371/journal.pone.0305827)
Supplement: S1 File — Research instruments questionnaires and topic guide. (DOCX) [file pone.0305827.s001.docx]

S1. Research instruments

S1A. Baseline Non-Financial questionnaire

1. Date:
2. Participant code:
3. Interviewer code:

Thank you for agreeing to take part in the study and for your time today. This survey should take about half an hour, but you have as much time as you need to answer the questions. This interview is completely voluntary, if we should come to any question that you do not want to answer, just let me know and we can continue on to the next question.

The survey includes 49 questions about your personal and household characteristics, financial situation, financial behaviour, health and wellbeing.

Your responses will be confidential. Anything you say that might mean that people would be able to identify you will not be shared with anyone outside the FinWell London research team.

At the end I will give you time to share any general comments or thoughts on the questions today.

Do you have any questions? Are you ready to begin?

**SECTION 1**

We will start with some questions about you and your household.

1. *How do you identify yourself in terms of gender?*

🞎 Male

🞎 Female

🞎 Other

1. What is your date of birth? *Day: ___ Month: ___ Year: _____*
2.
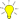
Which is your current employment situation?^[[1]](#footnote-1)^

*Employee in full time job (30 hours or more);*

*Employee in part time job (less than 30 hours);*

*Self-employed – full or part time;*

*Government supported training;*

*Unemployed and available for work;*

*Wholly retired from work;*

*Full time education (school, college, university);*

*Looking after family/home;*

*Permanently sick or disabled;*

*Casual work / cash in hand work;*

*Other (please specify)] [specify]*

1. Who else lives in this household with you? What are their sex, age, relationship to you, and occupation? Think about those who share a budget. Now I am going to ask you about each of them. Please include students who may be away at university / college or those currently in institutions but have this address as their main residence.

DO ANY OF THOSE HOUSEHOLD MEMBERS NEEDS LOOKING AFTER, ARE YOU THEIR CARER? ARE YOU A CARER OF SOMEONE ELSE OUTSIDE OF YOUR HOUSEHOLD?

Which is [NAME's] current employment situation?

|  | **Sex** | **Age** | **Relationship to Interviewee** | **Employment status** |
| --- | --- | --- | --- | --- |
| **1** |  |  |  |  |
| **2** |  |  |  |  |
| **3** |  |  |  |  |
| **4** |  |  |  |  |
| **5** |  |  |  |  |
| **6** |  |  |  |  |

1.
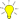
What is your ethnic group?

*British/English/Scottish/Welsh/Northern Irish*

*Irish*

*Gypsy or Irish Traveller*

*Any other White background*

*White and Black Caribbean*

*White and Black African*

*White and Asian*

*Any other mixed background*

*Indian*

*Pakistani*

*Bangladeshi*

*Chinese*

*Any other Asian background*

*Caribbean*

*African*

*Any other Black background*

*Arab*

*Any other ethnic group [specify]*

1. What is your country of origin? ______________
2. How long have you lived in the UK? Years_____
3.
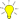
Do you have the feeling that your English has made interactions with people harder?

🞎 Completely agree

🞎 Sometimes

🞎 I couldn’t be sure

🞎 Not at all

1.
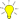
What is the highest level of education that you have completed?

🞎 Primary school or less

🞎 GCSEs, O Levels, CSE, School Certificate, Scottish Ordinary, Lower Certificate, 3/Foundations S Grade, Scottish Access 1-2, Scottish intermediate 2/Credit S Grade, Foundation GNVQ, Intermediate GNVQ, BTEC first certificate, SVQ/NVQ level 1-2, Level 1-2 vocational awards, IVQ certificate/Technician/Diploma, Level 1-2 International Introductory Awards

🞎 Vocational A-Levels, AVCE, BTEC National, Certificate/Diploma, City and Guilds SVQ/NVQ level 3, Level 3 vocational awards, IVQ Technician Diploma, IVQ Advanced Diploma, Level 3 International Awards

🞎 A-levels or Higher Certificate, Scottish Higher Certificate, International Baccalaureate

🞎 Nursing certificate, Teacher training, HE Diploma, Edexcel/B, Full technical certificate, BTEC HND/HNC, City and Guilds Licentiateship (LCGI), Graduateship (GCGI), Associateship (ACGI), Membership (MCGI), Master Professional, Diploma, Fellowship (FCGI), Higher Professional Diploma, SVQ/NVQ level 4-5, Level 4-5 vocational awards, IVQ Advanced Technician Diploma

🞎 3-4 year University, CNAA first Degree (BA, BSc., BEd., BEng.)

🞎 5 year University, CNAA first Degree (MB, BDS, BV etc)

🞎 Masters Degree, M.Phil, PGCE, PGDip, PGDE, PGCert, Post-Graduate Diplomas and Certificates

🞎 Ph.D, D.Phil or equivalent

🞎 Other (please specify): ___________________________

1. Have you received any training in financial management (budgeting/assessment of income and expenditure/record keeping/debt management/savings/ understanding of bank charges)?

🞎 No

🞎 Yes

*IF YES:*

1. Please give details of that training: (ie. Who provided it? How long was it? How did you fund it?)

**SECTION 2**

The next few questions are about health and wellbeing.

1.
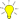
In general, would you say your health is:

🞎 Excellent

- Very good
- Good
- Fair
- Poor

1. Do you have any long-standing physical or mental impairment, illness or disability? By 'long-standing' we mean anything that is a chronic condition you are currently managing with medication or a medical problem that has troubled you over a period of at least 3 months.

- No 🞎 Yes

IF YES…

1. Please specify your (health) condition(s)?
2. Are you taking any medications for it? 🞎 No 🞎 Yes

IF YES…

- 1. could you specify? _________________________________________________

1.
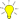
Does this/Do these health problem(s) or disability(ies) mean that you have substantial difficulties with any of the following areas of your life?

🞎 Mobility (moving around at home and walking)

- Lifting, carrying or moving objects
- Manual dexterity (using your hands to carry out everyday tasks)
- Continence (bladder and bowel control)
- Hearing (apart from using a standard hearing aid)
- Sight (apart from wearing standard glasses)
- Communication or speech problems
- Memory or ability to concentrate, learn or understand
- Recognising when you are in physical danger
- Your physical co-ordination (e.g. balance)
- Difficulties with own personal care (e.g. getting dressed, taking a shower)
- Other health problem or disability. Please specify: __________________

1.
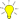
The following items are about activities you might do during a typical day. Does your **health now limit you** in these activities? If so, how much?
2. **Moderate** activities, such as moving a table, pushing a vacuum cleaner, bowling or playing golf

🞎 Yes, limited a lot 🞎 Yes, limited a little 🞎 No, not limited at all

1. Climbing **several** flights of stairs

🞎 Yes, limited a lot 🞎 Yes, limited a little 🞎 No, not limited at all

1. During the **past four weeks**, have you had any of the following problems with your work or other regular daily activities **as a result of your physical health**?
2. **Accomplished less** than you would like 🞎 No 🞎 Yes
3. Were limited in the **kind** of work or other activities 🞎 No 🞎 Yes
4. During the **past four weeks**, have you had any of the following problems with your work or other regular daily activities **as a result of any emotional problems** (such as feeling depressed or anxious)?
5. **Accomplished less** than you would like 🞎 No 🞎 Yes
6. Did work or other activities **less carefully than usual** 🞎 No 🞎 Yes

Are there any comments you would like to add about this section?

1.
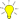
During the **past 4 weeks**, how much did **pain** interfere with your normal work (including both work outside the home and housework)?

🞎 Not at all

- A little bit
- Moderately
- Quite a bit
- Extremely

1.
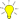
These questions are about how you feel and how things have been with you during the **past 4 weeks**. For each question, please give the one answer that comes closest to the way you have been feeling.

How much of the time during the **past 4 weeks**...?

|  | All of the time | Most of the time | A good bit of the time | Some of the time | A little of the time | None of the time |
| --- | --- | --- | --- | --- | --- | --- |
| 1. Have you felt calm and peaceful? | 🞎 | 🞎 | 🞎 | 🞎 | 🞎 | 🞎 |
| 1. Did you have a lot of energy? | 🞎 | 🞎 | 🞎 | 🞎 | 🞎 | 🞎 |
| 1. Have you felt downhearted and blue? | 🞎 | 🞎 | 🞎 | 🞎 | 🞎 | 🞎 |

1.
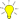
During the **past 4 weeks**, how much of the time has your **physical health or emotional problems** interfered with your social activities (like visiting friends, relatives, etc.)?

🞎 All of the time

- Most of the time
- A good bit of the time
- Some of the time
- A little of the time
- None of the time

1.
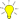
I am going to read five statements that you may agree or disagree with. Please indicate your level of agreement with each item using a 1 - 7 scale in which 7 means that you strongly agree with the statement, 6 - Agree, 5 - Slightly agree, 4 - Neither agree nor disagree, 3 - Slightly disagree, 2 – Disagree and 1 - Strongly disagree with the statement. Please be open and honest in your responding.

____ In most ways my life is close to my ideal.

____ The conditions of my life are excellent.

____ I am satisfied with my life.

____ So far I have gotten the important things I want in life.

____ If I could live my life over, I would change almost nothing.

1. Please indicate which statements best describe your overall quality of life at the moment for each of the five groups below.
   1. **Feeling settled and secure**

🞎 I am able to feel settled and secure in **all** areas of my life

🞎 I am able to feel settled and secure in **many** areas of my life

🞎 I am able to feel settled and secure in **a few** areas of my life

🞎 I am **unable** to feel settled and secure in **any** areas of my life

- 1. **Love, friendship and support**

🞎 I can have **a lot** of love, friendship and support

🞎 I can have **quite a lot** of love, friendship and support

🞎 I can have **a little** love, friendship and support

🞎 I **cannot** have **any** love, friendship and support

- 1. **Being independent**

🞎 I am able to be **completely** independent

🞎 I am able to be independent in **many** things

🞎 I am able to be independent in **a few** things

🞎 I am **unable** to be at all independent

- 1. **Achievement and progress*^[[2]](#footnote-2)^**

🞎 I can achieve and progress in **all** aspects of my life

🞎 I can achieve and progress in **many** aspects of my life

🞎 I can achieve and progress in **a few** aspects of my life

🞎 I cannot achieve and progress in **any** aspects of my life

- 1. **Enjoyment and pleasure**

🞎 I can have **a lot** of enjoyment and pleasure

🞎 I can have **quite a lot** of enjoyment and pleasure

🞎 I can have **a little** enjoyment and pleasure

🞎 I cannot have **any** enjoyment and pleasure

1. Any comments on this section, or anything you want to add? [If disabled, please record the condition and any other details provided such as since when the participant suffers the condition. Also record if any other household member has a disability or chronic condition.

**SECTION 3**

These questions are about health and finance.

1.
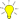
How much do your / your household’s health conditions affect your household’s finances?

- Not at all
- A little
- Some
- Very much
- A lot

1.
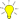
Now the other way round: How much does your / your household’s financial situation affect your household’s health?

- Not at all
- A little
- Some
- Very much
- A lot

1. In the past 3 months, how many times did you miss an entire work day because of problems with your physical or mental health? (Please include only days missed for your own health, not someone else’s health.) __________________________________________________________
2. During the past 12 months, was there any time when you or your household members needed medical care, but did not get it? 🞎 No 🞎 Yes 🞎 Don’t know
   1. IF YES, why? What did you do instead?
3. Any comments on this question, or anything you want to add? *(This box is also intended to clarify or further explore the reasons for replies in this section)*

**SECTION 4**

The next few questions are about risk.

I’m going to present you with a set of choices that relate to decisions when outcomes are uncertain.

There is no real money involved, and there are no actual financial consequences of your choices. This is purely for research, and all of your answers are confidential.

But I want you to think about the questions as if they were real choices and there was real money at stake, so take as much time as you need to make your choices.

The possibilities reflect 50-50 chances, as if a coin had been flipped:

1. *
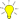
*If you were offered one from these 6 choices, which would you choose? Each circle represents a possibility with a 50% chance of either number occurring (as if determined by flipping a coin). There is no real money involved, but take your time to think about it as if it were an actual choice. Select only one circle.

**1)**  **2)**

**6)** **3)**

**5)**  **4)**

1. *
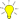
* Now, I’ve changed the amounts. If you were offered one from these 6 choices, which would you choose? Each circle represents a possibility with a 50% chance of either number occurring. Select only one circle.

**1) 2)**

**6) 3)**

**5) 4)**

1. *
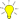
*If you were offered one from these 6 choices, which would you choose? Each circle represents a possibility with a 50% chance of either number occurring. The negative figures represent losses. Select only one circle.

**1) 2)**

**6) 3)**

**5) 4)**

1. Any comments on this section, or anything you want to add?

**SECTION 5**

The next questions concern getting money at different times. There is no real money involved, and there are no financial consequences of your choices. But I want you to think about the questions as if they were real choices and there was real money to be paid to you in cash. Take as much time as you need to make your choices.

1.
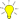
For each row, please select whether you would prefer OPTION A or OPTION B

| **Option A** | **Option B** | **A or B?** |
| --- | --- | --- |
| £53 guaranteed today | £56 guaranteed in one month |  |
| £49 guaranteed today | £56 guaranteed in one month |  |
| £45 guaranteed today | £56 guaranteed in one month |  |
| £42 guaranteed today | £56 guaranteed in one month |  |
| £35 guaranteed today | £56 guaranteed in one month |  |
| £28 guaranteed today | £56 guaranteed in one month |  |

1.
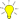
For each row, please select whether you would prefer OPTION A or OPTION B

| **Option A** | **Option B** | **A or B?** |
| --- | --- | --- |
| £53 guaranteed today | £56 guaranteed in 6 months |  |
| £49 guaranteed today | £56 guaranteed in 6 months |  |
| £45 guaranteed today | £56 guaranteed in 6 months |  |
| £42 guaranteed today | £56 guaranteed in 6 months |  |
| £35 guaranteed today | £56 guaranteed in 6 months |  |
| £28 guaranteed today | £56 guaranteed in 6 months |  |

1.
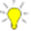
For each row, please select whether you would prefer OPTION A or OPTION B

| **Option A** | **Option B** | **A or B?** |
| --- | --- | --- |
| £53 guaranteed in 6 months | £56 guaranteed in 7 months |  |
| £49 guaranteed in 6 months | £56 guaranteed in 7 months |  |
| £45 guaranteed in 6 months | £56 guaranteed in 7 months |  |
| £42 guaranteed in 6 months | £56 guaranteed in 7 months |  |
| £35 guaranteed in 6 months | £56 guaranteed in 7 months |  |
| £28 guaranteed in 6 months | £56 guaranteed in 7 months |  |

1. Any comments on this section, or anything you want to add?

**SECTION 6
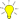
**

The next questions are about your ability to organize and predict the activities in your life. If you are unsure about how to answer a question, please give the best answer you can.

1. My life is organized

🞎 Strongly agree

🞎 Agree

🞎 Unsure

🞎 Disagree

🞎 Strongly disagree

1. My life is unstable

🞎 Strongly agree

🞎 Agree

🞎 Unsure

🞎 Disagree

🞎 Strongly disagree

1. My routine is the same from week to week

🞎 Strongly agree

🞎 Agree

🞎 Unsure

🞎 Disagree

🞎 Strongly disagree

1. My daily activities from week to week are unpredictable

🞎 Strongly agree

🞎 Agree

🞎 Unsure

🞎 Disagree

🞎 Strongly disagree

1. Keeping a schedule is difficult for me

🞎 Strongly agree

🞎 Agree

🞎 Unsure

🞎 Disagree

🞎 Strongly disagree

1. I do not like to make appointments too far in advance because I do not know what might come up

🞎 Strongly agree

🞎 Agree

🞎 Unsure

🞎 Disagree

🞎 Strongly disagree

**SECTION 7**

The next few questions are about trust.

1.
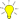
Is your neighbourhood a close-knit neighbourhood^[[3]](#footnote-3)^?

🞎 No

🞎 Yes

🞎 Don’t know

🞎 Somewhat

1.
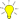
Do people in your neighbourhood share the same values?

🞎 No

🞎 Yes

🞎 Don’t know

🞎 Somewhat

1.
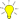
How often do you and other people in the neighbourhood ask each other advice about personal things such as child rearing or job openings?

🞎 Never

🞎 Rarely

🞎 Sometimes

🞎 Often

1.
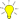
Do you feel safe walking alone in the area near your home?
   1. *During the day*

🞎 Completely safe

🞎 Fairly safe

🞎 Neither safe or unsafe

🞎 Fairly unsafe

🞎 Not safe at all

- 1. *During the night*

🞎 Completely safe

🞎 Fairly safe

🞎 Neither safe or unsafe

🞎 Fairly unsafe

🞎 Not safe at all

1. How many close friends would you say you have? Think about people who are not members of your family and you could rely on for help (family problems, financial, health). People you would resort to in a difficult situation.

*Please enter the number of people _________________*

1. Generally speaking, would you say that most people can be trusted or that you can’t be too careful in dealing with people?

🞎 You can’t be too careful

🞎 Most people can be trusted

🞎 Don’t know

1.
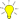
Please look carefully at the following list of organisations. For each of them, please say which, if any, you belong to and which, if any, you are currently doing unpaid work for?

|  | Do not belong | Belong to ONLY | Belong to AND volunteer |
| --- | --- | --- | --- |
| 1. Social club | 🞎 | 🞎 | 🞎 |
| 1. Sports club and/or team | 🞎 | 🞎 | 🞎 |
| 1. Community organisation – tenants association, community centre, neighbourhood campaign/watch etc. | 🞎 | 🞎 | 🞎 |
| 1. Church, Mosque or religious organisation | 🞎 | 🞎 | 🞎 |
| 1. Political party and/or pressure group | 🞎 | 🞎 | 🞎 |
| 1. School Parent Teacher Associations | 🞎 | 🞎 | 🞎 |
| 1. Support groups or welfare organisations | 🞎 | 🞎 | 🞎 |
| 1. Hobby or interest groups | 🞎 | 🞎 | 🞎 |
| 1. Pensioners club, lunch club | 🞎 | 🞎 | 🞎 |
| 1. Gym | 🞎 | 🞎 | 🞎 |
| 1. Other groups. Please specify: _____________ | 🞎 | 🞎 | 🞎 |

We have reached the end of the questionnaire. Do you have any comments on any of these questions, or anything you want to add?

Thank you for your time.

S1B. Baseline Financial questionnaire

1. Date:
2. Participant code:
3. Interviewer code:

Thank you for agreeing to take part in the study and for your time today. This questionnaire should take about **30 minutes**, but you have as much time as you need to answer the questions. Your participation is completely voluntary, if we should come to any question that you do not want to answer, just let me know to continue onto the next question.

The questionnaire includes **45** questions about your financial situation, financial management, financial knowledge and financial behaviour.

Your responses will be anonymised and treated confidentially. Anything you say that may make you identifiable to others will not be shared outside the FinWell London research team.

At the end, I will give you time to share any general comments or thoughts on the questions today.

Do you have any questions? Are you ready to begin?

**SECTION 1**

1. For this project you would be asked to keep records of your income and expenditure, do you already record household income and expenditure on a monthly basis?

🞎 No

🞎 Yes, household income

🞎 Yes, personal income

🞎 Other (please specify): _____________________________________________

1.
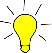
People organise their family finances in different ways, which of these ways comes closest to the way you organise yours?

🞎 I look after all the household’s money (except some personal spending money for my partner, if any)

🞎 My partner looks after all the household’s money (except my personal spending money, if any)

🞎 I am given a housekeeping allowance, my partner looks after the rest of the money

🞎 We share and manage our finances jointly

🞎 We keep our finances completely separate

🞎 Some other way - please specify: ____________________________________

🞎 Not applicable – live alone

1. Are you comfortable with reporting the income and expenditure of your household?

🞎 No

🞎 Yes, and will be able to estimate it accurately

🞎 Yes, but will only be able to give an approximate estimate

1. Do you separate the money for the household from that of the business?

🞎 No

🞎 Yes

1. Do you keep records of your sales and withdrawals?

🞎 No

🞎 Sometimes

🞎 Always

**SECTION 2**

The next few questions are about financial choices and knowledge.

1.
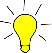
How strongly do you agree or disagree with the following statement?

Considering what you already know about personal finance, you could still benefit from some advice and answers to everyday financial questions from a professional.

🞎 Strongly agree

🞎 Somewhat agree

🞎 Somewhat disagree

🞎 Strongly disagree

🞎 Do not know

🞎 Refuse

1. Have you ever received financial advice?

🞎 Yes

🞎 No

*IF YES:* When? From which organisation/who? And what for?

1. How confident do you feel managing your money? Please answer on a scale of 0 to 10 where 0 is ‘not at all confident’, and 10 is ‘completely confident’ _______________
2. If the chance of getting a disease is 10 percent, how many people out of 1,000 would be expected to get the disease? ­­­­­­­­_______________
3. Let’s say you have £200 in a savings account. The account earns 10 percent interest per year. How much will you have in the account at the end of two years?

Answer in GBP : £____________

🞎 Do not know

🞎 Refuse

1. Annual percentage rate (APR) takes into account all fees attached to a loan.

🞎 True

🞎 False

🞎 Don’t know

1.
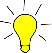
Which of the following credit card users is likely to pay the GREATEST amount in finance charges per year, if they all charge the same amount per year on their cards?

🞎 Jessica, who pays at least the minimum amount each month and more when she has the money

🞎 Vera, who generally pays off her credit card in full but occasionally, will pay the minimum when she is short of cash

🞎 Megan, who always pays off her credit card bill in full shortly after she receives it

🞎 Erin, who only pays the minimum amount each month

🞎 Don’t know

1.
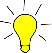
You will improve your creditworthiness by:

🞎 Visiting your local commercial bank

🞎 Showing no record of personal bankruptcies in recent years

🞎 Paying cash for all goods and services

🞎 Borrowing large amounts of money from your friends

🞎 Donating money to charity

🞎 Don’t know

**SECTION 3**

The next questions concern housing and assets.

1.
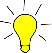
In which of these ways do you occupy your accommodation?

🞎 Owned by member of household with a mortgage/loan

🞎 Owned by member of household outright

🞎 Part rent/part mortgage

🞎 Rented from a housing association

🞎 Rented from housing association – please specify:

🞎 Rented from a private landlord

🞎 Living here rent free

🞎 Other – please specify:

*IF RENTING*

1. If you do not own your own home, why not?

🞎 Can’t afford to

🞎 Can’t obtain a mortgage

🞎 Bad time to buy

🞎 Other - please specify:

1.
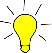
Is your current accommodation sufficient or insufficient for your current needs?

(This is just in terms of physical space only rather than about other dimensions of adequacy such as environment, noise, quality of maintenance, security, etc.)

🞎 More than sufficient

🞎 Sufficient

🞎 Insufficient

🞎 Very insufficient

*ONLY IF INSUFFICIENT OR VERY INSUFFICIENT*

- 1.
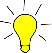
Are you prevented from moving home for any reason?

🞎 Yes- Can’t afford it

🞎 Yes- Council won’t rehouse me

🞎 Yes- Family responsibilities

🞎 Yes- Health reasons - please specify: _______________

🞎 Yes- Other reason -please specify: _______________

🞎 No

1. If your home is owned, please give an estimate of its market value (to the nearest £5,000):

_________________________________________________________________________

1. If your home is owned with a mortgage, how much is your monthly mortgage payment?:

_________________________________________________________________________

1. If your home is rented, how much is your monthly rental? : ________________________
2. In total, how many cars, vans, motorcycles or scooters are owned, or are available for private use, by you or members of your household? Include any company cars or vans available for private use.

Owned by you – please specify: _______________

Owned by other household members – please specify: _______________

**SECTION 4**

The next questions are about your income from employment, benefits, and other sources. We need to have information that is as detailed as possible.

|  | You | Your partner | Other adult |
| --- | --- | --- | --- |
| JOB 1 |  |  |  |
| Income from work (net) | £ | £ | £ |
| Income from work (gross) | £ | £ | £ |
| JOB 2 |  |  |  |
| Income from work (net) | £ | £ | £ |
| Income from work (gross) | £ | £ | £ |
| JOB 3 |  |  |  |
| Income from work (net) | £ | £ | £ |
| Income from work (gross) | £ | £ | £ |

1. What is your occupation? ______________________________________________________
2. How much do you take home per month from this job, after tax and National Insurance have been deducted (i.e. net pay)?
3. What was your total pay per month before any deductions for tax, national insurance, union dues and so on (including overtime, bonuses, commission or tips i.e. gross pay)?
4. What is his/her occupation? _____________________________________________________
5. Do you know how much does he/she takes home per month from this job, after tax and National Insurance have been deducted?
6. Do you know how much is his/her total pay per month before any deductions for tax, national insurance, union dues and so on (including overtime, bonuses, commission or tips)?
7. What is his/her occupation? _____________________________________________________
8. Do you know how much does he/she takes home per month from this job, after tax and National Insurance have been deducted?
9. Do you know how much is his/her total pay per month before any deductions for tax, national insurance, union dues and so on (including overtime, bonuses, commission or tips)?
10. About how much income after tax and other deductions {do you/your partner/other adult} take out of the business for use? (By income, I mean money drawn from the business which is used for personal, domestic, non-business use, in other words, what you have to live on).

1.
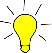
I'd like to talk about income from sources other than work. Are you {or your partner or any other adult in the household} receiving any benefits?

🞎 Yes

🞎 No

🞎 Don’t know

🞎 Refused

a. Which benefits do you {or your partner or any other adult in the household} receive?

b. How much do you {or your partner or any other adult in the household} receive for each?

c. How often do you {or your partner or any other adult in the household} receive it?

|  | **You** | **Your partner** | **Other Adult** | **Regularity** |
| --- | --- | --- | --- | --- |
| **Income-Related** |  |  |  |  |
| 1. Income Support |  |  |  |  |
| 1. Employment and support allowance (ESA) |  |  |  |  |
| 1. Working Tax Credit |  |  |  |  |
| 1. Child Tax Credit |  |  |  |  |
| 1. Jobseeker’s Allowance (JSA)   New style |  |  |  |  |
| 1. Jobseeker’s Allowance (JSA) Income based |  |  |  |  |
| 1. Jobseeker’s Allowance (JSA) 2. Contribution based |  |  |  |  |
| 1. Universal credit |  |  |  |  |
| 1. Housing Benefit |  |  |  |  |
| 1. Council Tax Benefit (Council tax reduction) |  |  |  |  |
| 1. Guardian’s Allowance |  |  |  |  |
| 1. Child Benefit |  |  |  |  |
| 1. Statutory Adoption Pay |  |  |  |  |
| 1. Maternity Allowance |  |  |  |  |
| 1. State Retirement Pension |  |  |  |  |
| 1. Pension Credit |  |  |  |  |
| 1. Statutory Maternity Pay |  |  |  |  |
| 1. Statutory Paternity Pay |  |  |  |  |
| 1. War Pension Scheme |  |  |  |  |
| 1. War Widow[er] Pension |  |  |  |  |
| 1. Bereavement Allowance, Bereavement Payment or Bereavement Support Payment |  |  |  |  |
| 1. Winter fuel payment |  |  |  |  |
| 1. Warm home discount |  |  |  |  |
| **Health-Related** |  |  |  |  |
| 1. Disability Living Allowance Care Component |  |  |  |  |
| 1. Disability Living Allowance Mobility Component |  |  |  |  |
| 1. Personal Independence Payment Mobility Component |  |  |  |  |
| 1. Personal Independence Payment Daily Living Component |  |  |  |  |
| 1. Industrial Injuries Disablement Benefit |  |  |  |  |
| 1. Carer’s Allowance |  |  |  |  |
| 1. Disablement Benefit |  |  |  |  |
| 1. Severe Disability Premium |  |  |  |  |
| 1. Statutory Sick Pay |  |  |  |  |
| 1. Armed Forces Compensation Scheme |  |  |  |  |
| 1. Attendance Allowance |  |  |  |  |
| 1. Constant Attendance Allowance |  |  |  |  |
| 1. Armed Forces Independence Payment |  |  |  |  |
| 1. Funeral Expenses Payment |  |  |  |  |
| 1. Exceptionally Severe Disablement Allowance |  |  |  |  |
| 1. Any other state benefit not already mentioned – please specify: |  |  |  |  |

1. Do you know the total value of the benefits received by your household per week?

£

1.
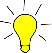
Do you {or any other member of your household} receive any other regular income or payment from any of these sources?

|  | **You** | **Your partner** | **Other adult** | **Regularity** |
| --- | --- | --- | --- | --- |
| **Formal** |  |  |  |  |
| 1. Employer-based pension |  |  |  |  |
| 1. Benefit from annuity, trust or covenant |  |  |  |  |
| 1. Maintenance payments |  |  |  |  |
| 1. Rent from property or sub-letting |  |  |  |  |
| 1. Benefit from accident/sickness scheme |  |  |  |  |
| 1. Investment income (e.g. dividends from shares/interest from savings) |  |  |  |  |
| 1. Grant (educational or otherwise) |  |  |  |  |
| **Informal** |  |  |  |  |
| 1. Receive money from other household members (as gifts or borrowed) |  |  |  |  |
| 1. Regular non-work income from any other source - please specify: |  |  |  |  |
| 1. Other - please specify: |  |  |  |  |

1.
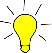
What is the total income (monthly/annually) coming into this household, after tax, including any benefits or pensions?

**Weekly Annual**

🞎 Nil Nil

🞎 Up to £69 Up to £3,599

🞎 £70-£129 £3,600-£6,599

🞎 £130-£189 £6,600-£9,599

🞎 £190-£249 £9,600-£12,599

🞎 £250-£309 £12,600-£15,599

🞎 £310-£379 £15,600-£19,199

🞎 £380-£479 £19,200-£23,999

🞎 £480-£599 £24,000-£29,999

🞎 £600-£719 £30,000-£35,999

🞎 £720-£859 £36,000-£42,999

🞎 £860-£999 £43,000-£49,000

🞎 £1000-£1499 £50,000-£74,999

🞎 £1500 or more £75,000 or more

**SECTION 5**

This section is about your bank accounts and savings.

1. Do you have a current account?

🞎 Yes

🞎 No

🞎 Don’t know

🞎 Refused

a. Which of these accounts do you have?

b. What is the name of the account provider?

c. How much have you saved in each?

d. How often do you save?

e. Do you get any interest rate from these accounts? If so, do you know how much?

|  | **Name of Provider** | **Size of Savings** | **Regularity of payment** | **Interest rate** |
| --- | --- | --- | --- | --- |
| **Bank Accounts** |  |  |  |  |
| 1. Current account |  |  |  |  |
| 1. Savings account |  |  |  |  |
| 1. Building society account |  |  |  |  |
| 1. Credit Union account |  |  |  |  |
| 1. Post Office account |  |  |  |  |
| 1. Individual Savings Account (ISA) |  |  |  |  |
| 1. Other (*please specify*) |  |  |  |  |

1. Do you have any shares, bonds or other investments?

🞎 Yes

🞎 No

🞎 Don’t know

🞎 Refused

1. Which type of investment do you have? *(eg. National Savings certificates and bonds / shares / insurance / bonds / employee shares and options / other investments?)*
2. What is the name of the provider?
3. What is the size of the investment?
4. How often do you get dividends / payments from the investment?
5. How much interest are you earning?

| **Type of investment** | **Name of provider** | **Size of investment** | **Regularity of payment from investment** | **Interest amount/rate** |
| --- | --- | --- | --- | --- |
|  |  |  |  |  |
|  |  |  |  |  |

1.
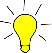
I’m going to read out a list of some of the ways in which people save money. Do you save money in any of these ways?

🞎 A Christmas Club or similar run by a local shop

🞎 Informally in a ‘*ménage’* or ‘money-go-round’ with work colleagues or friends

🞎 Putting money by in a jar or envelope (or somewhere else)

🞎 Asking a relative or friends to save or look after money for you

🞎 Lending money to friends or family as a way of saving

🞎 In conjunction with something you already pay e.g. loan/credit card/BrightHouse

🞎 Another way - please specify: ____________________________________________

🞎 None

🞎 Don’t know

🞎 Refused

1. How much do you have in savings in total in any of these ways *(sum to date)*?

£

1. Any comments on this section, or anything you want to add?

**SECTION 6**

The next few questions are about debt.

1. Have you ever been bankrupt?

🞎 Yes

🞎 No

🞎 Don’t know

1.
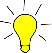
Do you have any debt?

🞎 Yes

🞎 No

🞎 Don’t know

🞎 Refused

1. Do you have any debts with any of the following lenders?
2. What is the name of the credit provider?
3. What was the reason for taking the loan?
4. What was the initial loan amount?
5. How much is remaining? What is the outstanding amount?
6. What is the amount and regularity of the instalments?
7. What is the interest rate?

|  | **Examples** | **Provider** | **Reason** | **Initial amount** | **Debt remaining** | **Instalment** | **Interest**  **rate** |
| --- | --- | --- | --- | --- | --- | --- | --- |
| **Formal/Regulated** |  |  |  |  |  |  |  |
| 1. Responsible providers | *Fair Finance, Scotcash* |  |  |  |  |  |  |
| 1. Internet/Payd-ay loans/High-Cost loans   (No Credit Check. Quick application procedure.) | *QuickQui-d, Amigo Loans, Oakam* |  |  |  |  |  |  |
| 1. Bank Loans   (Through a formal bank.) | *HSBC, Barclays, NatWest* |  |  |  |  |  |  |
| 1. Loan from your company or employer   (Anything going through company books.) | *Pay Advance* |  |  |  |  |  |  |
| 1. Credit Cards (Through a formal bank.) | *Barclayca-rd, Capital One, First Direct* |  |  |  |  |  |  |
| 1. Doorstep Loans   (Small regular payments at high interest rates and commonly flexible repayment schedules.) | *Provident (‘Provi’), Light Finance,* *First Choice* |  |  |  |  |  |  |
| 1. Store Cards (Credit cards to be used at certain shops - not ‘points’ cards.) | *Debenha-ms, Argos, New Look* |  |  |  |  |  |  |
| 1. Rent to Own/ Hire purchase/Week-ly payment store | *BrighHou-se, PerfectHo-me* |  |  |  |  |  |  |
| 1. Catalogue/ Mail order schemes | *Park Christma-s, Littlewoo-ds* |  |  |  |  |  |  |
| 1. Credit Union Loans | *London Capital Credit Union* |  |  |  |  |  |  |
| 1. Student loan | *Student Loans Company* |  |  |  |  |  |  |
| 1. Overdraft (Through formal bank.) | *HSBC, Barclays, Natwest, Lloyds* |  |  |  |  |  |  |
| **Informal/ Unregulated** |  |  |  |  |  |  |  |
| 1. Logbook Loan (Loan secured against vehicle.) | *Ramsdens, Varooma, Auto-Money* |  |  |  |  |  |  |
| 1. Loans from Friends or Family |  |  |  |  |  |  |  |
| 1. Individual Lenders |  |  |  |  |  |  |  |
| 1. Pawn Shop (Cash paid against material security for short time. May also issue postal orders/ non-bank guarantees.) | *Cash Converter-s, H&T Brixton, The Luxury Hut, Prestige* |  |  |  |  |  |  |
| 1. Other – please specify: |  |  |  |  |  |  |  |

1. Any comments on this section, or anything you want to add?

**SECTION 7**

This is the last section; the next questions are about unexpected events and financial experiences.

1. Has there been a particular event which has affected your household in the past year that has led to a worsening of your financial situation? For example, has anyone been ill or become unemployed?

🞎 Unemployment, redundancy, short time working

🞎 Ill health

🞎 Became pregnant, had a child

🞎 Family break up

🞎 Partner left

🞎 Death of member of household

🞎 Domestic violence

🞎 Other (please specify)

🞎 No

1. When did it occur?
2.
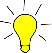
How often would you say you have been worried about money during the last few weeks?

🞎 Almost all the time

🞎 Quite often

🞎 Only sometimes

🞎 Never

1. If you had a financial emergency and needed £1000 in a hurry, what do you think you would do?

*_____________________________________________________________________________________________________________________________________________________________________________________________________________________________________________*

45. Before we end, I want to give you a chance to share any other thoughts about the kinds of issues we discussed today, or any connections to your own life and concerns.

Thank you very much for your time.

S1C. Final questionnaire

1. Date:
2. Participant code:
3. Interviewer code:

Thank you for agreeing to take part in the study and for your time today. This survey should take about half an hour, but you have as much time as you need to answer the questions. This interview is completely voluntary, if we should come to any question that you don't want to answer, just let me know to continue on to the next question.

The survey includes questions about your personal and household characteristics, financial situation, financial behaviour, financial management, financial knowledge, health and wellbeing.

Your responses will be confidential. Anything you say that might mean that people would be able to identify you will not be shared with anyone outside the FinWell London research team.

At the end I’ll give you time to share any general comments or thoughts on the questions today and the FinWell London project in general.

Do you have any questions?

**SECTION 1**

We will start with an update about your employment status.

1. Which is your current employment situation?

🞎 Employee in full time job (30 hours or more)

- Employee in part time job (less than 30 hours)
- Self-employed – full or part time
- Government supported training

🞎 Unemployed and available for work

- Wholly retired from work
- Full time education (school, college, university)
- Looking after family/home
- Permanently sick or disabled
- Casual work / cash in hand work
- Other (please specify)]

**SECTION 2**

The next few questions are about health and wellbeing.

1. In general, would you say your health is:

🞎 Excellent

- Very good
- Good
- Fair
- Poor

1. The following items are about activities you might do during a typical day. Does your **health now limit you** in these activities? If so, how much? *(show card)*
2. **Moderate** activities, such as moving a table, pushing a vacuum cleaner, bowling or playing golf

🞎 Yes, limited a lot 🞎 Yes, limited a little 🞎 No, not limited at all

1. Climbing **several** flights of stairs

🞎 Yes, limited a lot 🞎 Yes, limited a little 🞎 No, not limited at all

1. During the **past four weeks**, have you had any of the following problems with your work or other regular daily activities **as a result of your physical health**?
2. **Accomplished less** than you would like
   🞎 No 🞎 Yes

1. Were limited in the **kind** of work or other activities
   🞎 No 🞎 Yes
2. During the **past four weeks**, have you had any of the following problems with your work or other regular daily activities **as a result of any emotional problems** (such as feeling depressed or anxious)?
3. **Accomplished less** than you would like
   🞎 No 🞎 Yes
4. Did work or other activities **less carefully than usual**
   🞎 No 🞎 Yes

1. During the **past 4 weeks**, how much did **pain** interfere with your normal work (including both work outside the home and housework)?

🞎 Not at all

- A little bit
- Moderately
- Quite a bit
- Extremely

1. These questions are about how you feel and how things have been with you during the **past 4 weeks**. For each question, please give the one answer that comes closest to the way you have been feeling.

How much of the time during the **past 4 weeks**…

|  | All of the time | Most of the time | A good bit of the time | Some of the time | A little of the time | None of the time |
| --- | --- | --- | --- | --- | --- | --- |
| 1. Have you felt calm and peaceful? | 🞎 | 🞎 | 🞎 | 🞎 | 🞎 | 🞎 |
| 1. Did you have a lot of energy? | 🞎 | 🞎 | 🞎 | 🞎 | 🞎 | 🞎 |
| 1. Have you felt downhearted and blue? | 🞎 | 🞎 | 🞎 | 🞎 | 🞎 | 🞎 |

1. During the **past 4 weeks**, how much of the time has your **physical health or emotional problems** interfered with your social activities (like visiting friends, relatives, etc.)? *(show card)*

🞎 All of the time

- Most of the time
- A good bit of the time
- Some of the time
- A little of the time
- None of the time

1. I am going to read five statements that you may agree or disagree with. Please indicate your level of agreement with each item using a 1 - 7 scale:

   7 means that you strongly agree with the statement, 6 - Agree, 5 - Slightly agree, 4 - Neither agree nor disagree, 3 - Slightly disagree, 2 – Disagree and 1 - Strongly disagree with the statement.

   Please be open and honest in your responding.

____ In most ways my life is close to my ideal.

____ The conditions of my life are excellent.

____ I am satisfied with my life.

____ So far I have gotten the important things I want in life.

____ If I could live my life over, I would change almost nothing.

1. Please indicate which statements best describe your overall quality of life at the moment for each of the five groups below.
   1. **Feeling settled and secure**

🞎 I am able to feel settled and secure in **all** areas of my life

🞎 I am able to feel settled and secure in **many** areas of my life

🞎 I am able to feel settled and secure in **a few** areas of my life

🞎 I am **unable** to feel settled and secure in **any** areas of my life

- 1. **Love, friendship and support**

🞎 I can have **a lot** of love, friendship and support

🞎 I can have **quite a lot** of love, friendship and support

🞎 I can have **a little** love, friendship and support

🞎 I **cannot** have **any** love, friendship and support

- 1. **Being independent**

🞎 I am able to be **completely** independent

🞎 I am able to be independent in **many** things

🞎 I am able to be independent in **a few** things

🞎 I am **unable** to be at all independent

- 1. **Achievement and progress**

🞎 I can achieve and progress in **all** aspects of my life

🞎 I can achieve and progress in **many** aspects of my life

🞎 I can achieve and progress in **a few** aspects of my life

🞎 I cannot achieve and progress in **any** aspects of my life

- 1. **Enjoyment and pleasure**

🞎 I can have **a lot** of enjoyment and pleasure

🞎 I can have **quite a lot** of enjoyment and pleasure

🞎 I can have **a little** enjoyment and pleasure

🞎 I cannot have **any** enjoyment and pleasure

1. Any comments or anything you want to add?

**SECTION 3**

The next questions concern your finances and money worries.

1. What is the total income coming into this household, after tax, including any benefits or pensions?

£

**Weekly Annual**

🞎 Nil Nil

🞎 Under £60 Under £3000

🞎 £60-£119 £3000-£5999

🞎 £120-£199 £6000-£9,999

🞎 £200-£299 £10,000-£14,999

🞎 £300-£479 £15,000-£24,999

🞎 £480 or more £25,000 or more

1. Considering all of the sources of income coming into your household each month, would you say that your household income is regular and reliable?

🞎 Yes

🞎 No

🞎 Don’t know

1. How often would you say you have been worried about money during the last few weeks?

🞎 Almost all the time

🞎 Quite often

🞎 Only sometimes

🞎 Never

**SECTION 4**

The following questions concern social capital and trust.

1. How many close friends would you say you have? Think about people who are not members of your family and you could rely on for help (family problems, financial, health). *_________________*
2. Generally speaking, would you say that most people can be trusted or that you can’t be too careful in dealing with people?

🞎 You can’t be too careful

🞎 Most people can be trusted

🞎 Don’t know

1. Please look carefully at the following list of organisations *(show card)*. For each of them, please say which, if any, you belong to and which, if any, you are currently doing unpaid work for?

|  | Do not belong | Belong to ONLY | Belong to AND volunteer |
| --- | --- | --- | --- |
| 1. Social club | 🞎 | 🞎 | 🞎 |
| 1. Community group – choir, toddler group | 🞎 | 🞎 | 🞎 |
| 1. Women’s Institute | 🞎 | 🞎 | 🞎 |
| 1. Sports club/team | 🞎 | 🞎 | 🞎 |
| 1. Community organisation – tenants assoc, community centre, neighbourhood campaign, neighbourhood watch, etc. | 🞎 | 🞎 | 🞎 |
| 1. Church, Mosque or religious organisation | 🞎 | 🞎 | 🞎 |
| 1. Political party | 🞎 | 🞎 | 🞎 |
| 1. School Parent Teacher Associations | 🞎 | 🞎 | 🞎 |
| 1. Support groups or welfare organisations | 🞎 | 🞎 | 🞎 |
| 1. Hobby or interest groups | 🞎 | 🞎 | 🞎 |
| 1. Pensioners club, lunch club | 🞎 | 🞎 | 🞎 |
| 1. Other groups. Please specify: _____________ | 🞎 | 🞎 | 🞎 |

**SECTION 5**

This section concerns coping strategies.

1. If you had a financial emergency and needed £1000 in a hurry, what do you think you would do?

*________________________________________________________________________________________________________________________________________________________________________________________________*

*If the respondent replies ‘I couldn’t cope’, please record here:*

We have reached the end of the questionnaire. Do you have any comments on any of these questions, or anything you want to add?

Do you have any more general comments on the FinWell project?

Is there anything you think we should have talked about during the course of the project but haven’t?

Thank you for your time and for your participation in the project.

**S1D. Topic guide for the in-depth interviews**

**Finances:**

To start with could you tell me about your financial situation:

- Has money ever been an issue? Why?
- Has money ever caused you problems? Can you describe these?

Have there been occasions recently when you didn’t have enough money to get by?

- If so, what did you do?
- Why did you/ did you not approach banks or moneylenders or family/friends?
- How often did this happen?

What was this experience like? How did this make you feel?

**Finances and Health**

Thinking about your financial situation and your health. Do you think there is a connection between your financial situation and your health?

- Why do you think that?

Could you describe a situation in which your finances have affected your health?

- What happened? What would have helped you in this situation?
- [Depending on initial answer] Can you think of any positive/negative experiences?

Could you describe a situation in which your health has affected your finances?

- What happened? What would have helped you in this situation?
- [Depending on initial answer] Can you think of any positive/negative experiences?

You’ve previously talked about your different health conditions, such as (*provide examples that are known about respondents*) … is there anything related to your financial life that you think made your health worse? Why do you think that?

Still thinking about your own health, is there anything related to your financial life that would have:

- improved your health when you first became ill?
- stopped you from developing multiple health conditions?
- helped you to manage your illness(es) OR slowed down progression of your illnesses?

You’ve previously described that your children have health conditions. What do you think caused these health conditions? What do you think would have helped stopped your children from developing these health conditions?

- Thinking about your financial situation, do you see a connection between this and the health and well-being of your children? *If yes*: could you explain a bit more?

**Experience of responsible finance**:

From our previous discussions you’ve mentioned that you’ve received *XX* loans from a responsible lender.

How did you find out about them?

- Why did you decide to approach them (rather than another lender)?

How did you use the loan?

Can you describe to me the process/stages you went through before receiving a loan from XX?

- What did you have to do?
- What was this experience like? How did it make you feel?

Could you tell me about receiving the loan?

- How did receiving the loan make you feel?

How has the repayment process been?

- How do you feel about making repayments? Have you experienced any issues? Has anything helped you to make repayments?

Overall, how was your experience of using these loans?

- What did you like/dislike about the experience? Why?

How would you feel about approaching another lender now?

- Has that changed? Why do you feel this way?

**Responsible Finance and Health:**

Do you think receiving a loan from XX has impacted your life?

- What happened? What changed for you?
- How has it helped? Has it had any negative impact?
- How is your financial situation now? How do you feel about it? Has it changed?

How has receiving a loan affected you/your relationships with family/friends/community? financial situation? employment status?

- How does that make you feel? Has it enabled you to do anything you previously wouldn’t have been able to do?

A number of people interviewed so far have mentioned that getting a loan from XX has affected their health and wellbeing… what do you think about that?

- Why do you think that?
- In what ways do you think getting a loan could affect someone’s health and wellbeing? Why?
- Has your health and wellbeing ever been affected by getting a loan? Or using another financial product? In what way?

Do you think there is anything a lender could do that would benefit your health and wellbeing? Why?

How do you think your health affects your use of financial products and services?

- What works well? What doesn’t work well?

**End of interview**

Those are all the questions I have, but do you think there are things that I have missed which you think are important to mention?

Would you like to clarify anything?

Do you have any questions for me?

1. Choose most appropriate response in terms of hours worked, but also record any secondary form of work/employment in notes. [↑](#footnote-ref-1)
2. “I can” refers to what is available to you if you need it [↑](#footnote-ref-2)
3. A neighbour where everybody knows each other, looks out for each other. [↑](#footnote-ref-3)
