# Supplementary material for: Living on low-incomes with multiple long-term health conditions: A new method to explore the complex interaction between finance and health
Source: PLoS One. 2024 Jun 26;19(6):e0305827. doi: 10.1371/journal.pone.0305827 (PMC11207141; doi:10.1371/journal.pone.0305827)
Supplement: S2 File — The illustrative cases of Anya, Sofia, Luisa and Andrea. (DOCX) [file pone.0305827.s002.docx]

**S2. Illustrative Cases**

**Illustrative case 1: Anya**

Anya is a 54-year-old single mum who lives in a flat with her daughter in Brixton. She is currently managing three long-term health conditions and is registered as disabled. Anya’s needs are mostly to manage chronic obstructive pulmonary disease (COPD), and issues with her breathing – for which she needs oxygen. Her daughter (and carer) is now 23 years old and the main support to her mother, helping her to manage her conditions. Anya’s daughter is at university and also currently works part-time as a security assistant, but her income does not contribute much to the household.

Anya used to work as a cook in a nursery and, later, a day centre but she was forced to reduce her number of working days as her conditions became more and more severe. For Anya, financial life was easier when she could still work. She struggled with the careful budgeting required when living on benefits:

*“I found that when I was in employment, it was a lot better. I didn’t have the issues that I have now. I think because even though you get a set wage, when you get benefits, you have to work it out to a tee, what you prioritise more than one thing, and I think that’s where I struggle with my finances.”* (Anya)

Anya’s budgeting struggles were also challenged by unexpected cuts to her welfare payments. From our interview with Anya, these cuts emerged as particularly detrimental because they led her to stop her life insurance payments:

*“It got a bit more daunting when my benefits were cut. So, you know, looking at people who they say they take away … I was fortunate. They didn’t take everything away, but having that loss when you’ve got commitments in place, once you’ve got that, and then for some of it to be taken away, you try and balance everything out that you were doing. So, little things like paying my life insurance, which isn’t a lot a month, but still having to juggle it around to pay certain things, I lost that for about nine months, and then it was reinstated. So, that played a big part, again, with mental issues and my finances as well.*” (Anya).

Already finding it hard to prioritise essential expenses and managing two loans, Anya’s fridge suddenly broke. She did not have many options but to top up her credit union loan (Table 1.1). She had started using credit unions when she was still working and had worked hard to create a credit history that preserved her access to further loans in case she needed it. Although access to credit from her credit union was possible, this new debt put pressure on Anya. When we interviewed her, she told us:

“[If] your circumstances change once you’ve taken it, that can be a pressure of … “Well, how am I going to pay this back, because I’ve gone past the period where I can say take it back. I don’t need it anymore.” (Anya)

Anya’s mental health was assessed after the fridge broke and Anya took the credit union loan. Figure 1.1 shows the trend in both expenditure and mental health composite scale scores from SF12v1 questionnaires. These graphs suggest that Anya’s mental health worsened as a consequence of having to deal with the broken fridge; in month 2 Anya’s mental health reached its lowest point during the study period.

Table 1.1: Anya's monthly budget for month 2 (August 10 - September 9, 2019)

| **Source of funds (£)** | **1574** |  |  | **Use of funds (£)** | **1678** |
| --- | --- | --- | --- | --- | --- |
| Housing benefit | 408 |  |  | Rent | 408 |
| *Loan from Credit Union* | 500 |  |  | *New fridge* | 600 |
| PIP | 350 |  |  | Groceries | 161 |
| ESA | 301 |  |  | Gift to daughter | 100 |
| Research incentives | 15 |  |  | Council tax | 4 |
|  |  |  |  | Electricity bills | 48 |
|  |  |  |  | Mobile phone bill | 27 |
|  |  |  |  | Water bills | 12 |
|  |  |  |  | TV (license and on demand) | 39 |
|  |  |  |  | Household items | 49 |
|  |  |  |  | Personal care | 11 |
|  |  |  |  | Transport and petrol | 45 |
|  |  |  |  | Gambling | 40 |
|  |  |  |  | Takeaway | 25 |
|  |  |  |  | Charity | 10 |
|  |  |  |  | Personal Loan (Credit Union) | 43 |
|  |  |  |  | Personal Loan (Fair Finance) | 56 |

Figure 1.1: Anya’s monthly trends in expenditure and mental health

**Illustrative case 2: Sofia**

Sofia is 45 years old and arrived in the UK 20 years ago. She was first diagnosed with schizophrenia and, after a few years, she developed hypertension, diabetes, chronic back pain and depression. Her ill health limits her daily tasks and she struggles to find a job. She lives alone with four dependent children in a council house. She does not have support with childcare, and she cannot afford full-time nursery for her youngest son. Sofia has also two older sons outside the UK. Both of them suffer from mental disorders, and she supports them financially. Sofia is registered as disabled. Her income is entirely made of benefits including: weekly payments of housing benefit and child benefit, Jobseeker’s Allowance (JSA) every two weeks, and child tax credit and PIP every four weeks.

Sofia’s monthly income is around £2,500 after housing benefits. However, her income does not meet the basic needs of her household. Figure 2.1 shows Sofia’s attempts to manage, weekly, between her irregular income (blue line) and spikes in expenditures (red line) that include groceries, electricity and children’s need, among others.

Sofia was able to smooth her consumption from one week through a combination of debts, including formal and informal loans (bank overdrafts, affordable credit, budgeting loans, and loans from friends). During her in-depth interview, Sofia tells us: *“That's how I survive. I survive on the loans.”*.

Sofia was simultaneously managing 6 loans during the study, as well as water and electricity arrears (Table 2.1). We left her just before COVID-19 struck with liabilities close to £6,200.

During the study period, the additional loans she took out (Figure 2.1, w/c 30^th^ August 2019, spike in income, blue line) were meant to: (a) manage foreseen gaps between PIP scheduled payments to guarantee that groceries could be bought and bills could be paid (Figure 2.1, w/c 27^th^ of September 2019, drop in in the blue line); (b) children’s schooling needs (school uniforms) could be bought (Figure 2.1**,** w/c 30^th^ August and 6^th^ September 2019); (c) continue to support her sons living outside the UK throughout September and October 2019; and (d) budget for predicted expenses for immigration legal procedures in November and December 2019 (£1,000 in total). In this instance, Sofia told us:

“Right now I saved some money £1000 and gave to the solicitor £750 still I must give the Home Office, £2,400. I don't even have it; I have remained in £250 to give to the solicitor. I don’t have it. I must wait until I get my benefit or I go and borrow some more money”. (Sofia)

Despite Sofia’s ability to foresee upcoming expenses and manage challenging weeks, her health was worsening. When we met Sofia in September and October, she felt particularly anxious and faintish due to fluctuating sugar levels. Her financially-related anxiety and stress was perceived by Sofia as impacting on her diet. She felt physically nauseous, and she found it difficult to organise her chaotic life. She knew she needed help but felt that the system did not understand her needs. She felt she was being judged:

*“They can sanction you for stealing you know, what they say is because you keep on doing it over and over, they don’t think oh financially this woman is struggling. There's a lot of things going on, sick people who really genuinely sick but then it's nothing to do with your sickness it's to do with the way of life”*. (Sofia)

In our final meeting in December 2019, Sofia told us:

“I don’t make ends meet, even if I get money. My life is not moving forward. I’m just in one place, I can’t move. My kids want to go on holiday.” (Sofia)

Figure 2.1: Sofia’s household weekly income and expenditure (£)


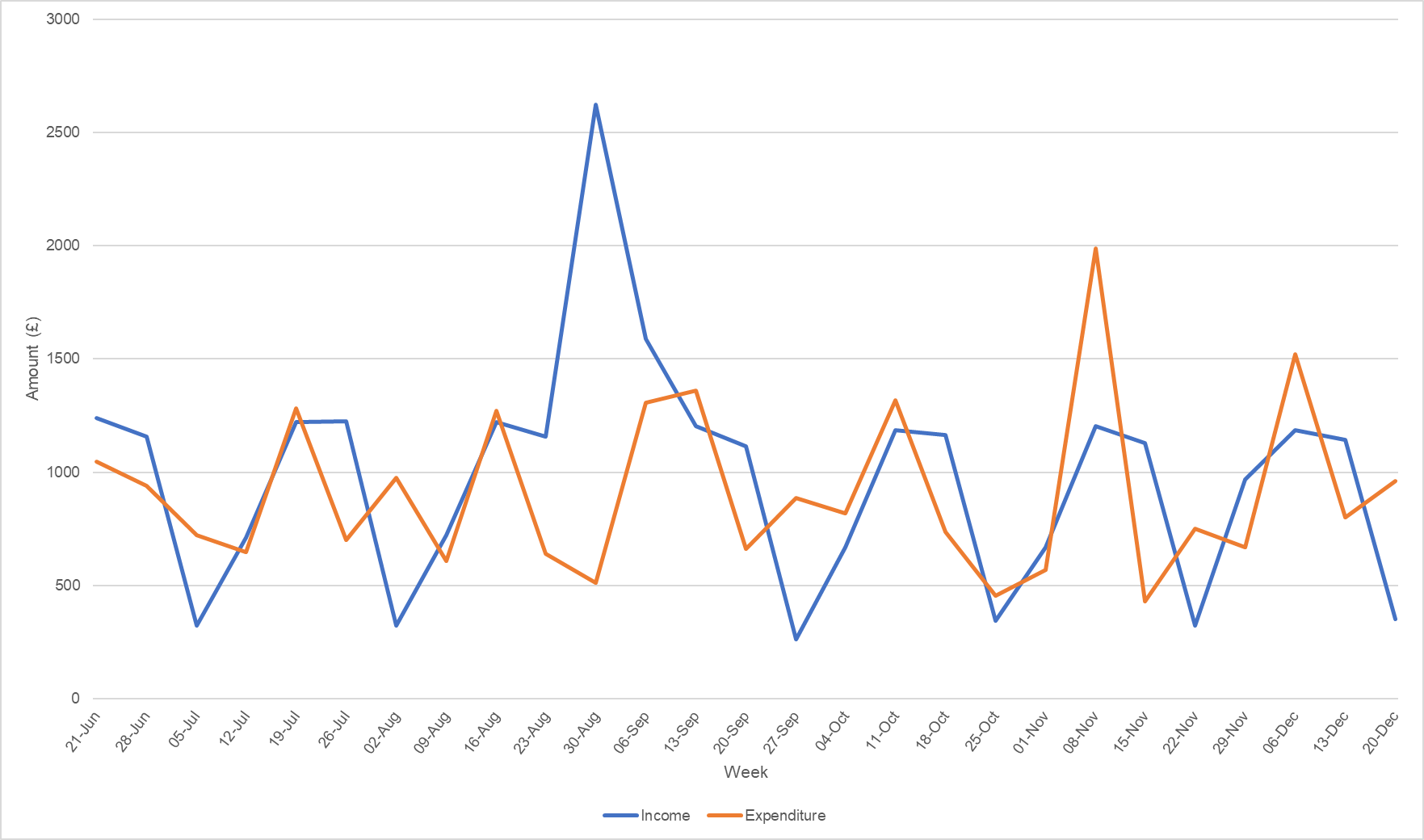


Table 2.1: Sofia’s monthly budget for month 6 (21 November – 20 December, 2019)

| **Source of funds (£)** | **4415** |  | **Use of funds (£)** | **4620** |
| --- | --- | --- | --- | --- |
| Child benefit | 1109 |  | Rent | 1048 |
| ESA | 692 |  | International phone credit | 25 |
| Housing benefit | 1048 |  | Mobile phone bills and internet | 62 |
| *Loan from Morses Club* | *300* |  | Pocket money to children | 100 |
| *Loan from neighbour* | *350* |  | Groceries | 226 |
| PIP | 896 |  | Netflix | 12 |
| Research incentives | 20 |  | Sky TV | 50 |
|  |  |  | Tobacco | 311 |
|  |  |  | Children expenses | 111 |
|  |  |  | Christmas gifts | 480 |
|  |  |  | Toiletries | 16 |
|  |  |  | Household items | 7 |
|  |  |  | Pet expenses | 25 |
|  |  |  | Account fees | 4 |
|  |  |  | Hairdressing (daughter) | 70 |
|  |  |  | Payment to solicitor (migration) | 500 |
|  |  |  | Hairdressing | 96 |
|  |  |  | Transports | 173 |
|  |  |  | Remittances to family | 550 |
|  |  |  | Electricity arrears | *140* |
|  |  |  | Water arrears | *15* |
|  |  |  | *Loan repayment to sister* | *55* |
|  |  |  | *Loan repayment to friend* | *20* |
|  |  |  | *Loan repayment to Fair Finance* | *264* |
|  |  |  | *Loan repayment to H&T* | *200* |
|  |  |  | *Loan repayment to Morses Club* | *60* |

**Illustrative case 3: Luisa**

Luisa is a 28-year-old mother of two young children (4 and 1) who lives with her husband Tony. The couple relocated from Portugal and have been living in the UK for the last 6 years. Luisa suffers from depression and her mental health deteriorated so much last year that she had to stop working. She used to work as a cleaner. Work played an important part in her social life and on the household’s finances. When we first meet her, she is feeling better and has started looking for a new job. However, the family has no help with childcare and the one-year-old only has 15-hours free nursery a week. Luisa is constrained: it is difficult to combine childcare responsibilities with low-pay work.

During the second month in the study, the family needs money and Luisa manages to find an informal cleaning job. It is little time and money but she is afraid that if she gets a more formal job the family will lose entitlement to universal credit, which is the most reliable income they have at the moment. She has to quit the job in month 3 as her anxiety was worsening because she was not yet fit to work. The household income (Figure 3.1) is not only low but also variable from month-to-month. Tony works on a zero-hour contract, and his salary does not always cover for essential expenses. At times when they are strapped for cash, they mostly rely on loans and gifts from Tony’s mum and Luisa’s aunty. Sometimes they are not in a position to help either, and then Luisa and Tony have to ask others such as Luisa’s boss. For example, the household budget on month 6 shows a £1,400 loan that the family got from Luisa’s boss. They urgently needed to rent a car whilst visiting their family in Portugal and they did not have enough cash. The loan was repaid 4 days later once they received a delayed Universal Credit payment. These informal debts are easier to renegotiate and some, particularly with Tony’s mum, never have to be repaid.

Figure 3.1: Luisa's household monthly income by type (in £; after rent)

However, the family spends around £500 monthly repaying loans and arrears, which are substantially less flexible. The first one, with Brighthouse, was used to buy much needed house appliances over a year ago. The second one is with the council. When Luisa stopped working they fell behind in their council tax payments and now they pay arrears twice every month. The third debt is with a mobile network provider and, finally, they have an overdraft with a commercial bank which they are trying to clear. The bills of the house are usually around £200. All these regular and important expenses leave them with little room to pay for the rest of the things they need, especially those that are unexpected or of high expense. Their income and expenses for months 4, 5, and 6 evidence the importance of servicing debt in their monthly budgets (see Tables 3.1-3.3).

Their income and expenditure is always very close which indicates that it is difficult for them to save **(**Figure 3.2). The months in which they do not receive any loans (1, 2, 4 and 5) they spend all the money that comes into the household, which does not leave much room to cope with any emergency expenditures.

When Luisa tells us about the connection between her depression and her finances she says that money uncertainty makes her stressed, noting that she has developed a rash in her skin. The rash comes and goes throughout the six months, depending on her stress levels and her income. The GP tells her she is somatising her mental issues.

Luisa also prefers daily visits to small, nearby stores rather than less frequent grocery shopping at a bigger supermarket, which could save her money. *“I prefer to pay this money every day”* she tells us. She adds that she does not have the mental space to plan her meals so she sometimes goes out for groceries several times a day.

Figure 3.2: Luisa’s household monthly income and expenditure (£)

When she is not doing well from her depression and anxiety, Luisa buys compulsively. She talks about *“the anxiety that wanting more than you need gives you (her)”*. She is trying to control this behaviour as it not only has financial implications for the household but also brings tensions to the relationship between the couple: *“my husband says I’ve spent £700 on amazon this year”*. The item ‘Miscellaneous (personal shopping)’ in the monthly budgets 4, 5 and 6 reflects this ‘compulsive’ behaviour (Tables 3.1-3.3).

Luisa is trying hard to improve her live and those of other members of her family. Apart from medical advice for her mental health and skin problems, she has also sought help from community groups in the Borough (mostly parenting groups which she tries to attend regularly) and is getting free psychotherapy services from a local organisation that her GP referred her to. After a period where she was finding hard to leave the house:

“*I just wanted to stay at home, next to the heating and watching TV. I’ve got anxiety with too many things in mind.*” (Luisa)

During the last research meeting she told us that in general she feels better and is making an effort to go out, attend group meetings and mingle with others: “*Otherwise I will fall again in the same circle as before*”. (Luisa)

When we last see her, Luisa is planning to further her education and get a secure and meaningful job. She is considering a £14,000 loan to pay for the course. It is a difficult decision to make; the pressure of the loan, the uncertainty of the household income, her delicate mental health and the lack of support with childcare are all factors that she needs to bear in mind.

Table 3.1: Luisa’ monthly budget for month 4 (19 October – 18 November, 2019)

| **Source of funds (£)** | **2338** |  | **Use of funds (£)** | **2415** |
| --- | --- | --- | --- | --- |
| Child benefits | 138 |  | Account fees | 8 |
| Employment income | 768 |  | Aeroplane ticket | 140 |
| Research incentives | 35 |  | Alcoholic drinks | 4 |
| Universal credit | 1277 |  | Charity | 15 |
| Loan from family and friends | 120 |  | Children expenses | 5 |
|  |  |  | Clothing | 82 |
|  |  |  | Concert | 15 |
|  |  |  | Contact lenses | 60 |
|  |  |  | Council arrears | 103 |
|  |  |  | Eating out | 84 |
|  |  |  | Electricity bill | 100 |
|  |  |  | Electronics | 163 |
|  |  |  | Gift to husband | 3 |
|  |  |  | Groceries | 325 |
|  |  |  | Hairdressing | 40 |
|  |  |  | Home insurance | 28 |
|  |  |  | Household items | 189 |
|  |  |  | Internet bills | 79 |
|  |  |  | Investment | 250 |
|  |  |  | IT insurance | 13 |
|  |  |  | Miscellaneous (personal shopping) | 97 |
|  |  |  | Mobile phone bill | 34 |
|  |  |  | Other health expenses | 12 |
|  |  |  | Overdraft | 200 |
|  |  |  | Overdraft fees | 22 |
|  |  |  | Personal care | 25 |
|  |  |  | Personal loan repayment (Brighthouse) | 50 |
|  |  |  | Personal loan repayment (O2) | 30 |
|  |  |  | Post office | 13 |
|  |  |  | Recorded music | 9 |
|  |  |  | Transports | 158 |
|  |  |  | TV license | 13 |
|  |  |  | TV show purchases | 17 |
|  |  |  | Water bills | 29 |

Table 3.2: Luisa’ monthly budget for month 5 (19 November – 18 December, 2019)

| **Source of funds (£)** | **2331** |  | **Use of funds (£)** | **2271** |
| --- | --- | --- | --- | --- |
| Child benefits | 138 |  | Accessories | 49 |
| Employment income | 900 |  | Account fees | 31 |
| Universal credit | 1293 |  | Alcoholic drinks | 4 |
|  |  |  | Charity | 15 |
|  |  |  | Children expenses | 58 |
|  |  |  | Clothing | 190 |
|  |  |  | Contact lenses | 33 |
|  |  |  | Council arrears | 253 |
|  |  |  | Eating out | 147 |
|  |  |  | Electricity bill | 50 |
|  |  |  | Gift to husband | 3 |
|  |  |  | Groceries | 318 |
|  |  |  | Hairdressing | 115 |
|  |  |  | Home insurance | 24 |
|  |  |  | Household items | 51 |
|  |  |  | Internet bills | 79 |
|  |  |  | IT insurance | 15 |
|  |  |  | Miscellaneous (personal shopping) | 152 |
|  |  |  | Mobile phone | 100 |
|  |  |  | Mobile phone bill | 26 |
|  |  |  | Overdraft | 200 |
|  |  |  | Personal care | 5 |
|  |  |  | Personal loan repayment (Brighthouse) | 50 |
|  |  |  | Personal loan repayment (O2) | 20 |
|  |  |  | Personal loan repayment to friend | 20 |
|  |  |  | Transports | 200 |
|  |  |  | TV license | 13 |
|  |  |  | TV show purchases | 17 |
|  |  |  | Video games | 4 |
|  |  |  | Water bills | 29 |

Table 3.3: Luisa’ monthly budget for month 6 (19 December 2019 - 18 January 2020)

| **Source of funds (£)** | **3445** |  | **Use of funds (£)** | **3123** |
| --- | --- | --- | --- | --- |
| Child benefits | 138 |  | Accessories | 13 |
| Employment income | 812 |  | Account fees | 25 |
| Loan from boss | 1400 |  | Alcoholic drinks | 6 |
| Mobile phone sold | 60 |  | Card fee | 7 |
| Universal credit | 1035 |  | Charity | 15 |
|  |  |  | Children expenses | 37 |
|  |  |  | Clothing | 33 |
|  |  |  | Contact lenses | 20 |
|  |  |  | Council arrears | 123 |
|  |  |  | Eating out | 302 |
|  |  |  | Electricity bill | 80 |
|  |  |  | Groceries | 175 |
|  |  |  | Hairdressing | 40 |
|  |  |  | Home insurance | 22 |
|  |  |  | Household items | 7 |
|  |  |  | Internet bills | 79 |
|  |  |  | IT insurance | 15 |
|  |  |  | Miscellaneous (personal shopping) | 66 |
|  |  |  | Other health expenses | 9 |
|  |  |  | Overdraft | 200 |
|  |  |  | Overdraft fees | 27 |
|  |  |  | Personal loan repayment (Brighthouse) | 50 |
|  |  |  | Personal loan repayment (O2) | 40 |
|  |  |  | Personal loan repayment (boss) | 1400 |
|  |  |  | Toiletries | 4 |
|  |  |  | Transports | 187 |
|  |  |  | TV license | 13 |
|  |  |  | TV show purchases | 23 |
|  |  |  | Unpaid bill fee | 23 |
|  |  |  | Video games | 53 |
|  |  |  | Water bills | 29 |

**Illustrative case 4: Andrea**

Andrea is a 44-year-old single woman living with no dependants and originally from Latin America. She moved to the UK in early 2018 to find a job and become financially stable. Before arriving in the UK, Andrea used to live in Spain, where she was registered as disabled due to several health conditions (arthritis.; gastrointestinal disorders; asthma; knee, shoulder, neck and spine injuries; epilepsy; gynaecological issues; thyroid problems; anxiety and depression). Andrea’s disability allowance in Spain was 380 euros a month, and her status of disabled person did not allow her to work. Andrea decided not to transfer her disability status to the UK. This strategy allowed her to find a part-time job in a cleaning company in London.

Being new to the UK and in part-time occupation, Andrea managed to find her first accommodation through a sub-let agreement. When we first met Andrea in summer 2019, she was living in single room in a shared flat, for which she used to pay £650 a month, plus £200 of supplementary costs if she wanted to host guests. She used to pay for it through Universal Credit (UC), which she accessed because she had been off-sick since April 2018, when she injured herself chronically at work while cleaning pools at a hospital. The conditions of her house and room were extremely adverse, and they worsened her already precarious health. She reported that her room and house conditions (humidity, mould and floods) exacerbated her asthma by making her have bad attacks at night, for which she needed more medication (Figure 4.1). For this reason, Andrea sought help from the council, which told her that she was eligible to access social housing.

During month 1 of this study, Andrea was told there were no social houses available yet. Andrea had to pay for a new month of private rent, and this challenged her health but also her finances, because the price charged by her landlord almost equalled her benefit payments, and she needed to ask for a Universal Credit budgeting advance to cope with illiquidity issues (Table 4.1).

Table 4.1: Andrea’s monthly budget for month 1 (July 5 - August 4, 2019)

| **Source of funds (£)** | **1329** |  | **Use of funds (£)** | **1310** |
| --- | --- | --- | --- | --- |
| Budgeting Advance (UC) | 348 |  | Account fees | 1 |
| Off-sick pay | 36 |  | Eating out | 14 |
| Universal Credit | 945 |  | Groceries | 83 |
|  |  |  | Household items | 13 |
|  |  |  | Internet | 11 |
|  |  |  | Loan repayment to a friend | 18 |
|  |  |  | Mobile phone bill | 21 |
|  |  |  | Netflix | 10 |
|  |  |  | Pension fund outside the UK | 180 |
|  |  |  | Rent | 850 |
|  |  |  | Storage space in Spain | 58 |
|  |  |  | Transports | 51 |

Figure 4.1: Andrea’s first accommodation during the study period


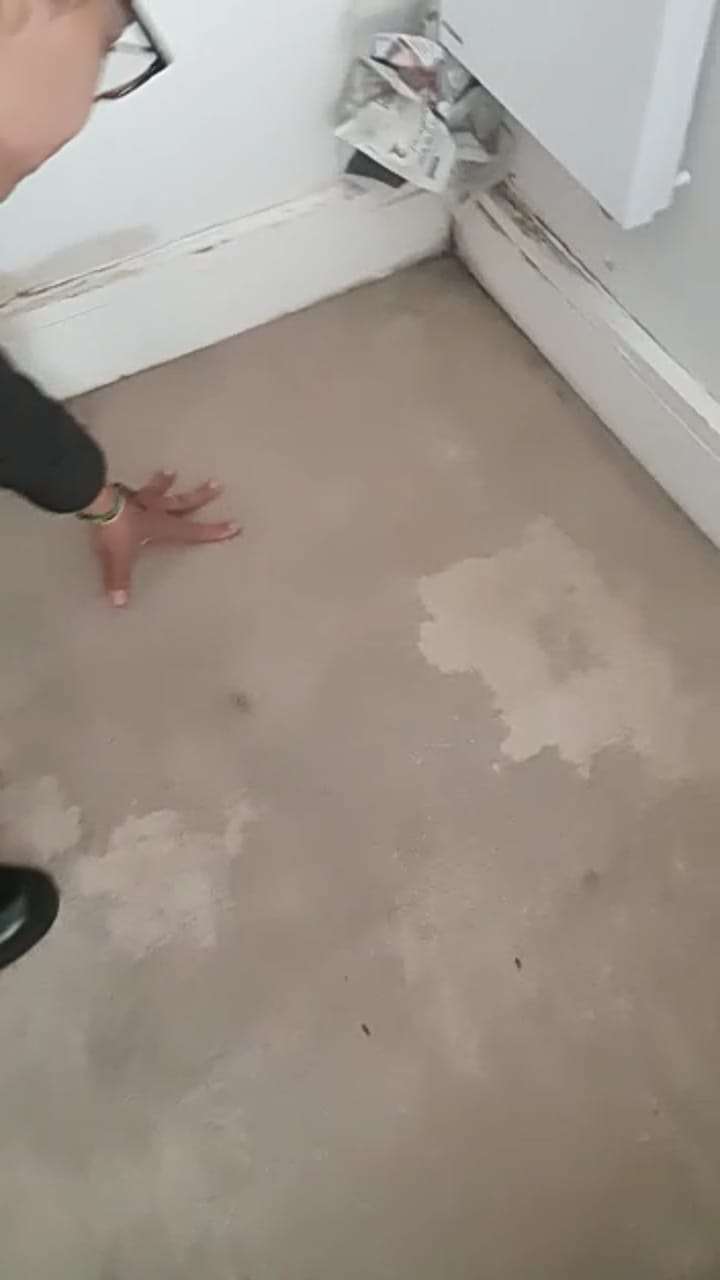

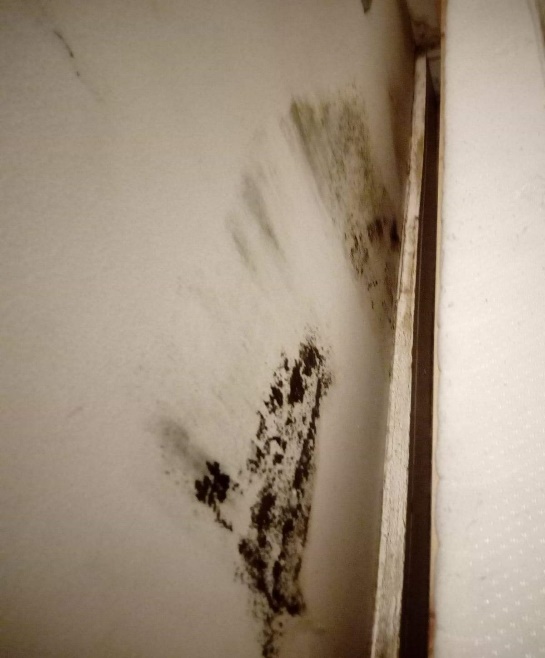

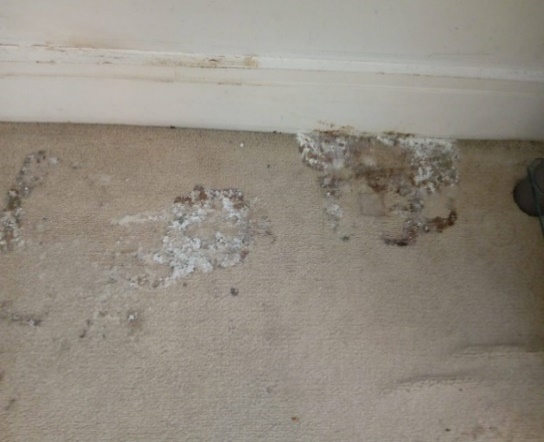

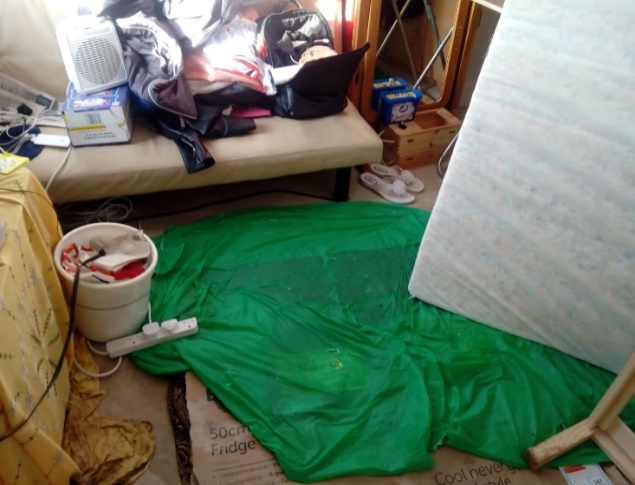

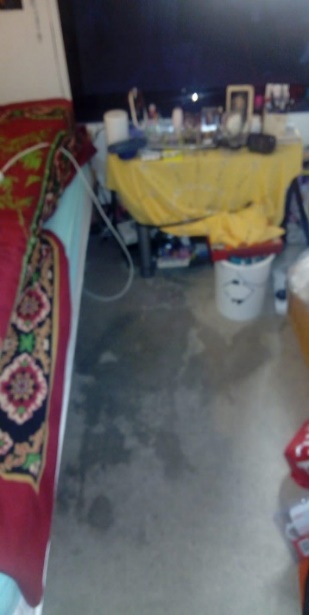


In month 2, Andrea was still waiting to be re-housed. This time, the council made a formal assessment of her accommodation, and they suggested to her not to pay for the rent, because the house was in a really bad condition. By skipping one month of rent, Andrea was able to save some money, find a new private accommodation and pay for the deposit and the first month of rent (Table 4.2).

Table 4.2: Andrea’s monthly budget for month 3 (September 5 - October 4, 2019)

| **Source of funds (£)** | **932** |  | **Use of funds (£)** | **1925** |
| --- | --- | --- | --- | --- |
| Off-sick pay | 36 |  | Account fees | 1 |
| Research incentives | 15 |  | Eating out | 8 |
| Universal Credit | 881 |  | Groceries | 117 |
|  |  |  | Household items | 43 |
|  |  |  | Optician | 241 |
|  |  |  | Loan repayment to friends | 58 |
|  |  |  | Mobile phone bill | 21 |
|  |  |  | Netflix | 10 |
|  |  |  | Pension fund outside the UK | 60 |
|  |  |  | Storage space in Spain | 58 |
|  |  |  | Transports | 92 |
|  |  |  | Clothing | 41 |
|  |  |  | Personal care | 10 |
|  |  |  | Rent new accommodation | 750 |
|  |  |  | Deposit new accommodation | 400 |
|  |  |  | Ankle wrap | 1 |
|  |  |  | Gift to a friend | 14 |

However, the money saved from month 2 was not enough to cover all the expenses in month 3. During the following months of this study, we observed that Andrea asked for several other loans from family and friends (worth £1830). Andrea’s UC was impacted by the scheduled repayments for the budgeting advance she asked for in month 1, and she still had to pay for a private rent because the council had not yet solved her housing situation. Andrea found the situation very frustrating and financially difficult. She felt unsupported by the council and she told us she was sent to visit the same houses multiple times, for which she was not eligible:

*“They only send me to places I’ve already been, as I don’t understand…”* (Andrea)

When we talked to her in December (month 6), she made it clear that she needed supplementary support to cope with her situation. Her health was comprised by the stress and lack of support with her housing case. Her employer had not assessed her case yet, and for that reason she could not try to go back to work and earn money. On that occasion, she had second thoughts about asking for disability status in the UK, and she told us:

*“Looking at pictures, how I was in Spain, yes, without a job because of the crisis, but I used to move around, I was studying, I dressed up nicely, had a social life…I arrived here and I haven’t been lucky … Days go by and I don’t know what I can claim, I need specific help, someone I can trust”*. (Andrea)

We met Andrea for the last time in February 2020. She was still waiting for her social housing to be fully solved. We left her before COVID-19 became a pandemic while she was living in a shared accommodation with around 12-14 people.
